# ProcCluster® and procaine hydrochloride inhibit the growth of *Aspergillus* species and exert antimicrobial properties during coinfection with influenza A viruses and *A. fumigatus in vitro*

**DOI:** 10.3389/fcimb.2024.1445428

**Published:** 2024-10-15

**Authors:** Sarah König, Josefine Schroeder, Thorsten Heinekamp, Axel A. Brakhage, Bettina Löffler, Beatrice Engert, Christina Ehrhardt

**Affiliations:** ^1^ Section of Experimental Virology, Institute of Medical Microbiology, Center for Molecular Biomedicine (CMB), Jena University Hospital, Jena, Germany; ^2^ Department of Molecular and Applied Microbiology, Leibniz Institute for Natural Product Research and Infection Biology – Hans-Knoell-Institute, Jena, Germany; ^3^ Department of Microbiology and Molecular Biology, Institute of Microbiology, Friedrich Schiller University, Jena, Germany; ^4^ Institute of Medical Microbiology, Jena University Hospital, Jena, Germany; ^5^ Inflamed Pharma GmbH, Jena, Germany

**Keywords:** *Aspergillus fumigatus*, aspergillosis, influenza-associated pulmonary aspergillosis, influenza virus, local anesthetics, procaine, ProcCluster®

## Abstract

**Introduction:**

Influenza-associated pulmonary aspergillosis is associated with high mortality rates and limited treatment options. The current standard practice involves treating each pathogen separately. However, the use of antifungal drugs can lead to serious side effects, and the presence of triazole-resistant *Aspergillus* strains can complicate antifungal therapy. In addition, drug-resistant influenza viruses are becoming an increasing concern in clinics. A drug that affects fungal and viral propagation could overcome these disadvantages. Thus, we conducted a study to examine the antifungal and antiviral properties of ProcCluster® and procaine hydrochloride (HCl), which are prodrugs derived from the local anesthetic procaine.

**Methods:**

Conidia of different *A. fumigatus* strains, *A. flavus* and *A. terreus* were treated with the test substances in a human cell-free system and antifungal properties were analyzed either by fluorescence microscopy or absorption measurements. Changes in metabolic activity and intracellular Ca^2+^ distribution during treatment of *A. fumigatus* with ProcCluster® were observed using fluorescence microscopy. In addition, antifungal and antiviral properties of ProcCluster® and procaine HCl were investigated during *in vitro* coinfection of lung epithelial cells with *A. fumigatus* and influenza A viruses (IAV). Analysis was performed by fluorescence microscopy, standard plaque assay and Western blot assay.

**Results:**

Both substances inhibited the growth of the fungus, even when applied after germination or in the presence of purified IAV particles. ProcCluster® remained effective against triazole-resistant *A. fumigatus* strains. However, the addition of CaCl_2_ reversed the antifungal effect, indicating that ProcCluster® inhibited fungal growth by disrupting fungal Ca^2+^ homeostasis. Furthermore, *in vitro* studies showed that ProcCluster® and procaine HCl reduced the pathogen load of IAV and *A. fumigatus* during coinfection. Finally, the combination of ProcCluster® with the antiviral drug favipiravir exhibited increased antipathogenic activity, particularly against IAV replication.

**Discussion:**

This research highlights ProcCluster® and procaine HCl as substances with anti-infective properties against various pathogens.

## 1 Introduction

Aspergillosis, most commonly caused by the species *Aspergillus fumigatus,* and to a lesser extent by *A. flavus*, *A. terreus* and *A. nidulans*, belongs to the most frequent fungal lung infection in humans. The opportunistic pathogen *A. fumigatus* typically occurs as a saprobe on decaying vegetation and soils, playing an important role in carbon and nitrogen recycling. By releasing its conidia into the air, *A. fumigatus* can reach the alveoli of the human lungs and, under certain conditions, cause invasive infections (Latge and Chamilos, 2019). In particular, immunocompromised patients, such as those undergoing hematopoietic stem cell or solid organ transplantation, are at high risk of developing invasive pulmonary aspergillosis (IPA) (Ledoux and Herbrecht, 2023). With mortality rates ranging between 25 and 60%, IPA remains a life-threatening disease in these high-risk groups (Neofytos et al., 2018; Pardo et al., 2019). In addition to neutropenia and other immunosuppressive conditions, IPA is known to complicate other diseases, such as influenza virus infections (van de Veerdonk et al., 2017; Schauwvlieghe et al., 2018).

Influenza viruses cause seasonal epidemics, resulting in 3–5 million severe cases and up to 650,000 fatalities annually worldwide (Iuliano et al., 2018). Influenza A viruses (IAV) in particular pose a major threat to public health because they are capable of causing pandemics that can result in millions of deaths, as seen for the Spanish flu in 1918/19 (Taubenberger and Morens, 2006). However, in most cases, patients recover easily from a flu and severe disease progression is often associated with secondary infections by bacteria or fungi. Coinfections with fungi are often related to species of the genus *Aspergillus* (van de Veerdonk et al., 2017; Schauwvlieghe et al., 2018; Bartley et al., 2022). To date, the molecular mechanisms of coinfections with influenza viruses and *Aspergillus* species have rarely been studied. Studies on mice revealed increased fungal burden, body weight loss and higher mortality in the presence of IAV and *A. fumigatus* compared to infection with either pathogen alone (Tobin et al., 2020; Seldeslachts et al., 2021a). Our own research, using an *in vitro* coinfection model, has shown that IAV-particles can bind to the surface of *A. fumigatus* and induce fungal growth (König et al., 2024). However, in the presence of *A. fumigatus,* viral titers of IAV and virus-related host cell responses were reduced (König et al., 2024). Nonetheless, clinical studies have demonstrated an increased mortality rate compared to that of pure influenza virus infection (Schauwvlieghe et al., 2018). Although early diagnosis and treatment of fungal infections can improve the outcome of influenza-associated pulmonary aspergillosis (IAPA) (van de Veerdonk et al., 2017), treatment options are limited.

So far, treating each pathogen individually seems to be the gold standard (Vanderbeke et al., 2018). The triazoles voriconazole and isavuconazole are recommended as first line therapies against IPA (Patterson et al., 2016; Ullmann et al., 2018). Triazoles inhibit the production of ergosterol, a component of the fungal cell membrane, resulting in growth inhibition. Although they are potent antimycotics with activity against various fungi, triazoles are associated with many adverse effects, including hepatotoxicity and cardiac complications (Wang et al., 2010; Chai et al., 2022). Triazoles also interact with cytochrome P450, an enzyme involved in the biotransformation of drugs, which can lead to drug-drug-interactions (Czyrski et al., 2021). Furthermore, serum concentrations of triazoles, especially voriconazole, can vary from patient to patient. Therefore, drug monitoring is recommended during triazole treatment (Ullmann et al., 2018). In addition to adverse events, resistance to available antifungal drugs may complicate IPA therapy (van der Linden et al., 2011; Lestrade et al., 2019).

Currently, three antiviral drug classes are described for the treatment of IAV infections: (I) neuraminidase inhibitors,(II) CAP-dependent endonuclease inhibitors and (III) M2-inhibitors (Duwe et al., 2021). However, reports of resistance to antiviral drugs are emerging and driving the development of new antiviral agents (Renaud et al., 2011; Abed and Boivin, 2017; Lampejo, 2020). One promising new drug is favipiravir, a polymerase inhibitor, which has already been approved in Japan (Furuta et al., 2017). However, substances that target viral structures are always at risk of inducing new resistances. To circumvent this problem, novel antiviral strategies focus on inhibiting virus-supportive cellular factors or enhancing antiviral defense mechanisms (Ludwig, 2009).

As eukaryotes, fungal and human cells share a number of similar cellular factors and signaling pathways. The most prominent example is the mitogen-activated protein kinase (MAPK) cascade, which is involved in various regulatory processes in fungi (May et al., 2005; Zhao et al., 2007). Another important process in cells is Ca^2+^ homeostasis. In *A. fumigatus*, activation of Ca^2+^-dependent pathways regulate fungal cell growth, virulence and stress tolerance (Cramer et al., 2008; de Castro et al., 2014; Li et al., 2024). Thus, substances that affect human cellular factors may also influence homologous factors in *A. fumigatus*.

Recently, we identified host-directed antiviral properties against SARS-CoV-2 and IAV for the ester-type local anesthetics (LAs) procaine prodrugs ProcCluster®, also known as procaine hydrogen carbonate, which is stabilized by NaCl, and procaine hydrochloride (HCl) (Häring et al., 2021, 2023, 2024). Both drugs have altered properties compared to procaine. In particular, ProcCluster® is characterized by an almost physiological pH and good water solubility, whereas procaine has a basic pH and is soluble in organic solvents. These improved properties enable multiple applications of ProcCluster®, such as inhalation or oral administration (Häring et al., 2024). ProcCluster® and procaine HCl reduce viral titers of IAV and SARS-CoV-2, for example, by inhibiting phospholipase activity or by affecting the release of progeny viruses (Häring et al., 2023, 2024). Furthermore, LAs are known for their antimicrobial functions and have been described to act as antifungal agents against *Candida* and *Aspergillus* species, probably by interfering with fungal Ca^2+^ homeostasis (Rodrigues et al., 2000, 2006).

Therefore, we investigated the antimycotic effects of ProcCluster® and procaine HCl against *A. fumigatus* and other *Aspergillus* species. Since both drugs are known to possess antiviral properties, we further analyzed their effects during coinfection with IAV and *A. fumigatus in vitro*.

## 2 Materials and methods

### 2.1 Conidia harvest

The following *A. fumigatus* strains were used: green fluorescent protein (GFP)-expressing *A. fumigatus* (parental strain: ATCC 46645) (Lother et al., 2014), *A. fumigatus* wild type (wt; ATCC 46645), *A. fumigatus* CEA10 (CBS 144.89), *A. fumigatus* Af293 and the azole-resistant strains C7-NRZ-2016-121 (C7) and C11-NRZ-2017-040 (C11). C7 and C11 are clinical isolates from IPA patients and are resistant to voriconazole, itraconazole and posaconazole (Barber et al., 2021). Azole-resistance of C7 is based on TR_34_/L98H resistance mechanism, while the genetic base of azole-resistance in C11 is unknown (Barber et al., 2021). In the following, the GFP-expressing *A. fumigatus* was used, unless otherwise stated. In addition to *A. fumigatus*, other *Aspergillus* species were tested, namely *A. flavus* (JMRC318) and *A. terreus* (i402). All *A. fumigatus* strains were grown in Sabouraud-2% dextrose broth (Sigma-Aldrich, Germany) at 37°C. After 7 days, the fungal mycelium with formed conidia was transferred to a fresh tube and vortexed together with phosphate-buffered saline (PBS) (Carl Roth, Germany) with 0.01% Tween20 (Carl Roth, Germany) (PBS-Tween20). The other *Aspergillus* species were grown on Sabouraud-2% glucose agar (Carl Roth, Germany) for 7 days at 37°C. The conidia were then detached from the mycelium using a cell scraper and PBS-Tween20. For all *Aspergillus* species and strains, the conidia-rich supernatant was filtered through a 30 µm strainer (Miltenyi Biotec, Germany), centrifuged (4,400 × g, 5 min), washed again and resuspended in PBS-Tween20.

### 2.2 Cell culture

The lung epithelial cell lines Cancer lung-3 (Calu-3, ATCC HTB-55™) and A549 cells (ATCC CCL-185™) were cultured in Dulbecco’s Modified Eagle’s Medium high glucose w/stable glutamine w/sodium pyruvate (DMEM; anprotec, Germany) supplemented with 10% fetal calf serum (FCS; anprotec, Germany) at 37°C and 5% CO_2_. Human bronchial epithelial primary cells (HBEpCs; PromoCell, Germany) were cultured in airway epithelial cell growth medium (EC; PromoCell, Germany) (37°C, 5% CO_2_) and used for up to five subcultures. Madin-Darby Canine Kidney (MDCK; ATCC CCL-34™) cells were cultured in Minimum Essential Medium w/Earle’s salts, w/L-glutamine (MEM; anprotec, Germany) supplemented with 10% FCS (37°, 5% CO_2_).

### 2.3 Virus culture

The influenza virus strain A/Puerto Rico/8/34 (H1N1, kind gift of Stephan Ludwig, Muenster, Germany) was propagated in MDCK cells with Panserin 401 (PAN-Biotech, Germany) supplemented with 0.25 µg ml^-1^ TPCK-Trypsin (Thermo Fisher Scientific™, USA) for 2 days (37°C, 5% CO_2_). Virus-rich supernatants were centrifuged (4,400 × g, 10 min, 4°C) and filtered through a 0.45 µm membrane (VWR®, USA). The virus harvest was either used in cell culture experiments or subjected to a purification process for use in host cell-free experiments. For the latter, 1500 µl virus suspension was applied to 400 µl of a 20% sucrose cushion and centrifuged for 90 min at 20,000 × g and 4°C. The supernatant was carefully removed and the pellet was resuspended in PBS_INF_ (PBS supplemented with 1 mM MgCl_2_, 0.9 mM CaCl_2_ and 0.2% bovine serum albumin (BSA)).

### 2.4 Drugs

ProcCluster®, provided by the inflamed pharma GmbH (Germany), and procaine HCl (Chongqing Southwest No.2 Pharmaceutical Factory Co.,Ltd., China) were dissolved in H_2_O. Favipiravir (T-705) was purchased from Selleckchem (Germany) and voriconazole from Dr. Friedrich Eberth Arzneimittel (Germany). The latter two drugs were dissolved in dimethyl sulfoxide (DMSO; Sigma-Aldrich, Germany).

### 2.5 Quantitative measurement of fungal growth in a human cell-free environment

Conidia of *A. fumigatus* were seeded on coverslips in 24-well plates (5×10^6^ ml^-1^) and were either treated with 0.31–10 mM of ProcCluster® or procaine HCl, solvent (H_2_O) or 0.2 µg ml^-1^ voriconazole for 10 h (37°C, 5% CO_2_) diluted in DMEM with 10% FCS. To examine the antifungal effect of ProcCluster® after germination of *A. fumigatus*, conidia were grown for 2–8 h (37°C, 5% CO_2_) and then treated with 2.5 mM ProcCluster®. The solvent was added at time 0. To investigate the effects of ProcCluster® during coincubation of *A. fumigatus* and IAV particles, conidia were treated with ProcCluster® (2.5 mM) or solvent in the presence or absence of purified IAV particles (5 × 10^7^ IAV per sample). All samples were incubated for 10 h (37°C, 5% CO_2_). Subsequently, samples were fixed in 3.7% formaldehyde (Sigma-Aldrich, Germany) for 1 h at room temperature (RT) and coverslips were mounted (Dako, USA) on glass microscope slides. Images were taken with an Axio Observer Z1 (Zeiss, Germany) using a Plan Apochromat 20/0.8 objective lens (Zeiss, Germany), ApoTome.2 (Zeiss, Germany) and an Axiocam 503 mono (Zeiss, Germany). For image acquisition, Zen2.6 software (Zeiss, Germany) was used. Image processing and quantification of fungal size were performed using the software particle counter (https://www.github.com/chko90/particleCounter). The software measures the area of each GFP-expressing fungi shown in the image and was used in a previous study (König et al., 2023).

### 2.6 FUN™1 vital dye staining of *A. fumigatus*

FUN-1 staining was first invented to visualize the metabolic activity of yeasts, such as *Saccharomyces cerevisiae*, under fluorescent light (Millard et al., 1997). Several publications have shown that this fluorophore also works in other fungi, including *A. fumigatus* (Lass-Flörl et al., 2001; Balajee and Marr, 2002). FUN-1 is taken up by the fungus and diffuses as green-yellow dye throughout the cytoplasm. Due to metabolic action, the dye is transported into vacuoles and converted to red cylindrical intravacuolar structures (CIVS). Nonviable cells do not metabolize the dye. Therefore, the cells still appear as equally green-yellow-colored fungi (Millard et al., 1997). Because of its unique properties, FUN-1 is a suitable dye for investigating the antifungal efficacy of various substances (Lass-Flörl et al., 2001; Pina-Vaz et al., 2001; Balajee and Marr, 2002). To examine the cell viability of *A. fumigatus*, conidia of *A. fumigatus* wt were seeded on coverslips in 24-well plates (5×10^6^ ml^-1^) and grown for 8 h (37°C, 5% CO_2_) in DMEM + 10% FCS. Subsequently, the hyphae were treated with 5 mM ProcCluster® or solvent and incubated for an additional 1.5 h. *A. fumigatus* was then incubated with 10 µM FUN™1 (Thermo Fisher Scientific, USA) together with 5 mM ProcCluster® or solvent diluted in water containing 2% glucose and 10 mM Na-HEPES (pH 7.2) for another 30 min (37°C, 5% CO_2_). Finally, the hyphae were fixed in 3.7% formaldehyde (30 min, RT) and the coverslips were wet-mounted on glass microscopy slides. Image acquisition was performed immediately. Information about image acquisition and processing can be found in section 2.5. As objective lens, Plan Apochromat 40/0.95 (Zeiss, Germany) was used.

### 2.7 Absorption measurements to analyze fungal growth after 48 h

The antimycotic effect of ProcCluster® and procaine HCl was also tested for a longer time period (48 h). For this purpose, conidia of various *A. fumigatus* isolates and other *Aspergillus* species were seeded in 96-well plates and treated with 0.31–10 mM inhibitor or solvent. Further, all *A. fumigatus* isolates were treated with 0.01–1 µg ml^-1^ voriconazole, to investigate their sensitivity to voriconazole. To examine the mechanism of action of ProcCluster® in more detail, conidia of *A. fumigatus* were treated with 10, 25 or 50 mM CaCl_2_ and 2 mM ProcCluster® or solvent or with 25 mM NaCl and KCl with or without 25 mM CaCl_2_ and 2 mM ProcCluster® or solvent. After 48 h (37°C, 5% CO_2_), absorption was measured at 620 nm using a plate reader (FLUOstar® Omega, BMG Labtech, Germany) (Kaiserer et al., 2003).

### 2.8 Intracellular Ca^2+^ staining and microscopy

The staining method used to investigate intracellular Ca^2+^ levels in filamentous fungi was previously described by Nair et al. and the protocol was adjusted to the requirements of the present study (Nair et al., 2011). Conidia of *A. fumigatus* wt were seeded on coverslips in 24-well plates (5×10^6^ ml^-1^) and grown for 8 h (37°C, 5% CO_2_) in FluoroBrite™ DMEM (Thermo Fisher Scientific, USA) containing 10% FCS and 1 mM CaCl_2_. Subsequently, the hyphae were treated with 5 mM ProcCluster®, solvent or 0.2 µg ml^-1^ voriconazole and incubated for an additional hour. Then, *A. fumigatus* was stained with 5 µM Calbryte™ 520 AM (AAT Bioquest, USA) and 5 µg ml^-1^calcofluor white (Sigma-Aldrich, Germany), in the presence of 5 mM ProcCluster®, solvent or 0.2 µg ml^-1^ voriconazole and incubated again for 1 h. The fungi were washed twice with medium and then were fixed in 3.7% formaldehyde (30 min, RT). Finally, the coverslips were wet-mounted on glass microscopy slides, and images were acquired immediately. Information about image acquisition and processing can be found in section 2.5.

### 2.9 Coinfection of lung epithelial cells with IAV and conidia of *A. fumigatus*

Recently, we established a coinfection model to investigate the pathogen-host and pathogen-pathogen interaction between lung epithelial cells, IAV and *A. fumigatus* (König et al., 2024). This model was used here to investigate the effect of ProcCluster® and procaine HCl during coinfection. Calu-3 and A549 cells were grown on 12-well plates (for standard plaque assay and Western blot analysis) or on coverslips in 24-well plates (for microscopy studies). Calu-3 cells were seeded at a concentration of 0.5 × 10^6^ cells per well (12-well plate) or 0.25 × 10^6^ cells per well (24-well plate), 36 h prior to infection. A549 cells were seeded with a concentration of 0.25 × 10^6^ cells per well (12-well plate) or 0.125 × 10^6^ cells per well (24-well plate), 24 h prior to infection. Cells were washed with PBS and either infected with IAV diluted in PBS_INF_ at a multiplicity of infection (MOI) of 0.5 or only with PBS_INF_ (control) for 30 min (37°C, 5% CO_2_). Subsequently, the supernatant was removed and exchanged with fresh DMEM_INF_ (DMEM supplemented with 1 mM MgCl_2_, 0.9 mM CaCl_2_, 0.2% BSA and 0,16 µg ml^-1^ TPCK-Trypsin) containing 2.5 mM ProcCluster®, procaine HCl or solvent. Simultaneously to drug treatment, cells were coinfected with conidia of *A. fumigatus* (10 MOI) or left uninfected. Samples were incubated for an additional 10 h (37°C, 5% CO_2_). HBEpCs were seeded on coverslips in 24-well plates (0.25 × 10^6^ cells per well; 36 h prior to coinfection) and treated in the same way as described above, except for the use of 5 MOI IAV and EC_INF_ (EC supplemented with 1 mM MgCl_2_, 0.9 mM CaCl_2_, 0.2% BSA and 0.16 µg ml^-1^ TPCK-Trypsin).

### 2.10 Combination treatment with ProcCluster® and favipiravir during IAV and *A. fumigatus* coinfection

Calu-3 cells were seeded on coverslips in 24-well plates (0.25 × 10^6^ cells per well, 36 h prior to infection). Cells were washed with PBS and infected with IAV (0.5 MOI in PBS_INF_) for 30 min (37°C, 5% CO_2_). Subsequently, the supernatant was removed and exchanged with DMEM_INF_ containing the indicated concentrations of ProcCluster® and favipiravir alone or in combination. Simultaneously to drug treatment, cells were coinfected with conidia of *A. fumigatus* (10 MOI). Samples were incubated for additional 10 h (37°C, 5% CO_2_). The supernatants were stored for standard plaque assay, which is described in the following section (2.11). To analyze fungal growth, samples were prepared and analyzed as described in section 2.5. Possible synergistic or additional effects were analyzed using the online tool SynergyFinder Version 3.0 (https://synergyfinder.fimm.fi) (Ianevski et al., 2022). The synergistic score was calculated using the zero interaction potency (ZIP) model (Ianevski et al., 2022).

### 2.11 Standard plaque assay

The supernatants of the experiments described in section 2.9 and 2.10 were used to determine infectious viral titers by standard plaque assay (Karakus et al., 2018). In brief, MDCK cells were seeded in 6-well plates (1.75 × 10^6^ cells per well, 24 h prior to infection). Cells were washed with PBS, infected with serially diluted virus (diluted in PBS_INF_ containing 0.5 µg ml^-1^ voriconazole) and incubated for 30 min at 37°C and 5% CO_2_. After adsorption, the supernatant was aspired and cells were covered with 2 ml of soft agar and incubated for 3 days (37°C, 5% CO_2_). Soft agar contains MEM (Gibco™, USA), 0.9% agar (Oxoid, UK), 0.01% DEAE-Dextran (Fine Chemicals, Sweden), 0.2% BSA (Carl Roth, Germany), 0.2% NaHCO_3_ (Gibco™, USA), 0.25 µg ml^-1^ TPCK-trypsin (Thermo Fisher Scientific, USA) and 0.5 µg ml^-1^ voriconazole. Plaque forming units (PFU) were determined by counting plaques upon neutral red-staining (Sigma-Aldrich, Germany).

### 2.12 Western blot analysis

Protein expression was investigated by Western blot analysis. For this purpose, cells were lysed with Triton lysis buffer (20 mM Tris-HCl (pH 7.4), 137 mM NaCl, 10% glycerol, 1% Triton-X-100, 2 mM EDTA, 50 mM glycerophosphate, 20 mM sodium pyrophosphate) containing proteinase inhibitors (0.2 mM Pefabloc® (Sigma-Aldrich, Germany), 5 µg ml^-1^ aprotinin (Carl Roth, Germany), 5 µg ml^-1^ leupeptin (Sigma-Aldrich, Germany), 1 mM sodium vanadate (Sigma-Aldrich, Germany) and 5 mM benzamidine (Sigma-Aldrich, Germany)) for 30 min at 4°C. Lysates were cleared via centrifugation (20,000 × g, 10 min, 4°C), adjusted equally by the Bradford method, supplemented with 5% Laemmli buffer (10% SDS, 50% glycerol, 25% 2-mercaptoethanol, 0.02% bromophenol blue, 312 mM Tris-HCl (pH 6.8)) and incubated for 10 min at 95°C. Samples (25 µl) and marker proteins (PageRuler prestained protein ladder, Thermo Fisher Scientific, USA) were loaded onto 10% SDS-PAGE. The separated proteins were blotted on 0.2 µm nitrocellulose-membranes. The membranes were then blocked for 1 h with 2% milk (Carl Roth, Germany) and incubated overnight with primary antibodies (1:1000): mouse anti-extracellular-signal regulated kinase (ERK2; sc-1647, Santa Cruz, USA), mouse anti-GFP (sc-9996, Santa Cruz, USA), rabbit anti-IAV H1N1 hemagglutinin (HA; PA5-20712, Thermo Fisher Scientific, USA) and rabbit anti-polymerase base 1 (PB1; GTX125923, GeneTex, USA). For visualization, horseradish peroxidase (HRP)-conjugated goat anti-mouse IgG (926-80010, LI-COR® Bioscience, Germany) or HRP-conjugated goat anti-rabbit IgG (926-80011, LI-COR® Bioscience, Germany) were used. Membranes were incubated in Pierce® ECL Western Blotting Substrate (Thermo Fisher Scientific, USA) and developed with the Fusion^©^FX6.Edge (Vilber Lourmat, Germany). Protein levels were quantified using the gel analysis software Bio-1D (version 15.08c; Vilber Lourmat, Germany). All signals were normalized to the ERK2 signal.

### 2.13 Fluorescence microscopy

Samples of the experiment described in section 2.9 were fixed in 3.7% formaldehyde for 1 h at RT, permeabilized with 0.01% Triton-X-100 for 20 min and blocked with 10% normal goat serum (Linaris, Germany) for another hour. Subsequently, mouse anti-IAV nucleoprotein (NP) primary antibodies (MCA400, Bio-Rad, USA) were added (1:200) for 1 h at RT. AlexaFluor®647-conjugated donkey anti-mouse IgG (A-31571, Invitrogen, USA) and bisbenzimide H33342 trihydrochloride (Hoechst 33342; 1:5000; BioLegend, USA) were added for another hour. Finally, the coverslips were mounted on glass microscope slides. For image acquisition, processing and quantification of fungal growth, refer to section 2.5.

### 2.14 Statistical analysis

Statistical analysis was performed using Prism 8 (Graphpad Software, USA). The following statistical assays were performed: (I) one-way analysis of variance (ANOVA) followed by Dunnett’s multiple comparison test, comparing the mean of all conditions with one control condition; (II) one-way ANOVA followed by Tukey’s multiple comparison, comparing the mean of all conditions to each other and (III) one sample t-test, comparing the mean of one condition to a hypothetical value, for example 100% or 1. The corresponding statistical test for each experiment is described in the figure legends.

## 3 Results

### 3.1 ProcCluster® inhibits the growth of *A. fumigatus* even after germination in a human cell-free environment

Antimicrobial activity had already been described for some LAs. Therefore, ProcCluster® and procaine HCl were initially investigated for their antifungal activity against *A. fumigatus* in a human cell-free system.

**Figure 1:**
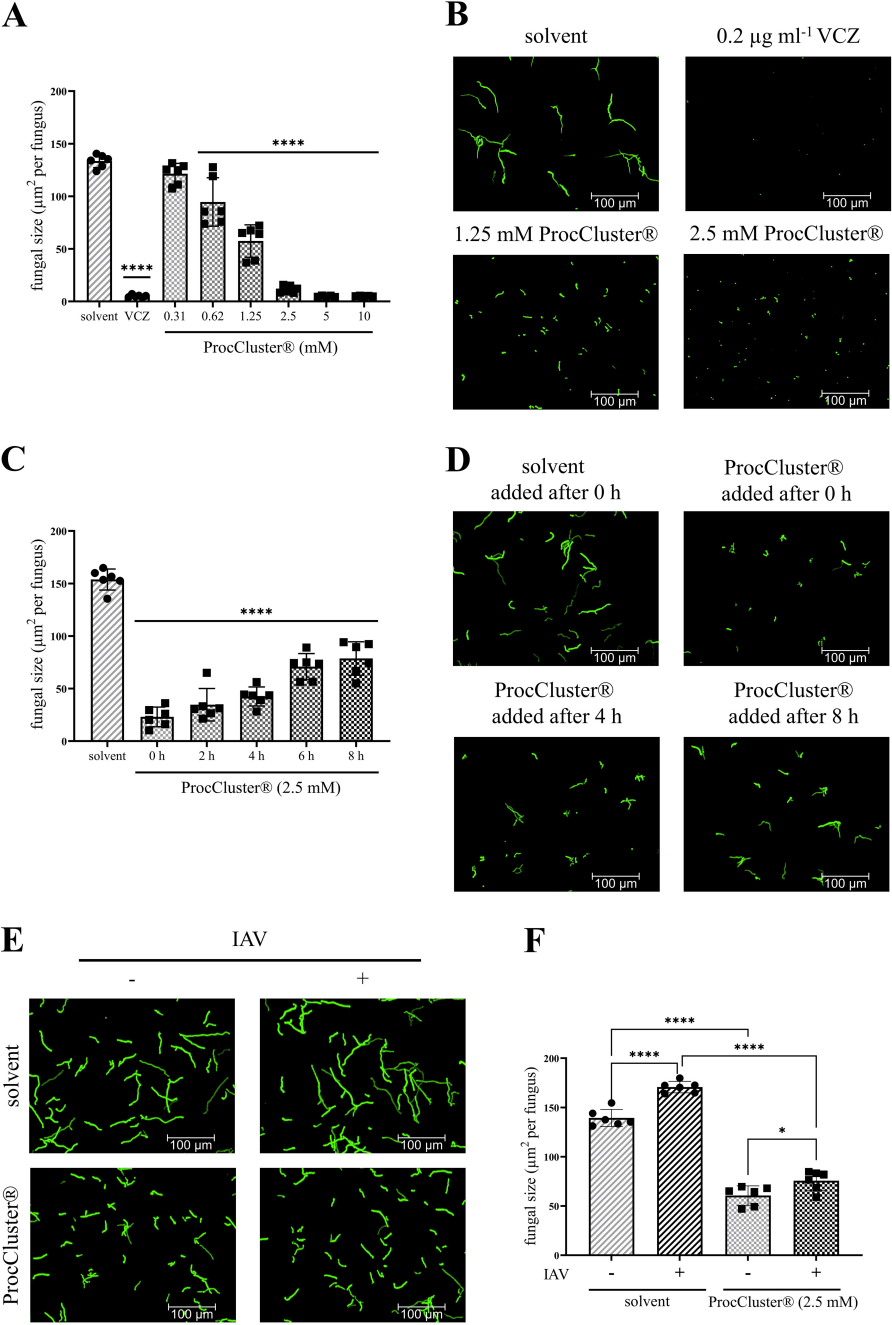
In the presence of ProcCluster® the growth of *A*. *fumigatus* is inhibited in a human cell-free environment. **(A, B)** Conidia of *A*. *fumigatus* were incubated with the indicated concentrations of ProcCluster®, solvent (H_2_O) or 0.2 µg ml^-1^ voriconazole (VCZ) for 10 h and growth of the fungus was quantified based on fluorescence microscopy. **(A)** The quantification of fungal size is given as µm^2^ per fungus. **(B)** Selected fluorescence images are shown as representative examples. **(C, D)** The effect of ProcCluster® to inhibit the progressive growth of *A*. *fumigatus* was tested for different addition times. **(C)** The graph shows the quantified fungal growth after a total incubation time of 10 h. ProcCluster® (2.5 mM) was added at the indicated time points and the solvent was added at time 0. **(D)** Fluorescence images show GFP-expressing fungi treated with solvent or with ProcCluster® after 0, 4 or 8 h of growth. **(E, F)** Finally, the antifungal effect of ProcCluster® was tested on influenza A virus (IAV)-induced hyphal growth. Therefore, the conidia of *A*. *fumigatus* were treated with 2.5 mM ProcCluster® or solvent. At the same time, purified IAV particles (5 × 10^7^ IAV per sample) were added to the fungus. GFP-expressing *A*. *fumigatus* was visualized **(E)** by fluorescence microscopy and quantified **(F)**. **(B, D, E)** All immunofluorescent images were taken using an Axio Observer.Z1 microscope (Zeiss) at 20 × magnification. Scale bars represent 100 µm. Images show one representative example of three independent experiments. **(A, C, F)** Diagrams show the mean (± SD) of the results of three independent experiments, including duplicates. Statistical significance was evaluated by one-way analysis of variance (ANOVA) followed by Dunnett’s multiple comparison test, comparing the mean of all samples to the solvent-treated sample **(A, C)**, or by one-way ANOVA followed by Tukey’s multiple comparison, comparing the means of all samples to each other **(F)** (****p ≤ 0.0001, *p ≤ 0.05).

Conidia were grown in the presence of different concentrations of ProcCluster®, solvent or 0.2 µg ml^-1^ voriconazole (VCZ). After 10 h, the samples were fixed and analyzed by fluorescence microscopy. The area of the green fluorescent fungi was then quantified and displayed as the unit µm^2^ per fungus. Significantly reduced growth of *A. fumigatus* is already visible at concentrations higher than 0.62 mM. At a concentration of 2.5 mM, the fungal size decreased to less than 10% of the measured size of the solvent-treated fungi. Higher concentrations of ProcCluster® inhibited growth, which was comparable to that of the VCZ-treated samples (**Figures 1A, B**). Treatment with the same concentrations of procaine HCl resulted in similar but less pronounced reductions (**Supplementary Figures 1A, B**).

Next, *A. fumigatus* was treated with 2.5 mM ProcCluster® after 2, 4, 6 or 8 h of cultivation. Fungal growth was determined after a total incubation time of 10 h (**Figures 1C, D**). The later ProcCluster® was added, the higher the fungal growth. Nevertheless, fungal growth was significantly lower at each time point tested compared to the solvent-treated control. Even when ProcCluster® was added for the last 2 h of incubation (8 h), the fungal growth was reduced to almost half the size of the solvent-treated sample (**Figure 1C**).

As we have recently shown that IAV particles bind to the surface of *A. fumigatus* and thereby induce fungal growth, we also tested whether ProcCluster® can efficiently reduce the growth of *A. fumigatus* in the presence of IAV. Therefore, conidia were incubated for 10 h with or without purified IAV particles (5 × 10^7^ IAV per sample) and 2.5 mM ProcCluster® or solvent. As expected, the presence of IAV particles in the solvent-treated control significantly increased the growth of *A. fumigatus*. However, ProcCluster® was still able to inhibit the fungal growth, regardless of the presence of the virus (**Figures 1E, F**).

In summary, ProcCluster® efficiently inhibited the growth of *A. fumigatus*, even when germination of the fungus had already begun or during IAV-induced fungal growth.

### 3.2 The viability of *A. fumigatus* is reduced in the presence of ProcCluster®

**Figure 2:**
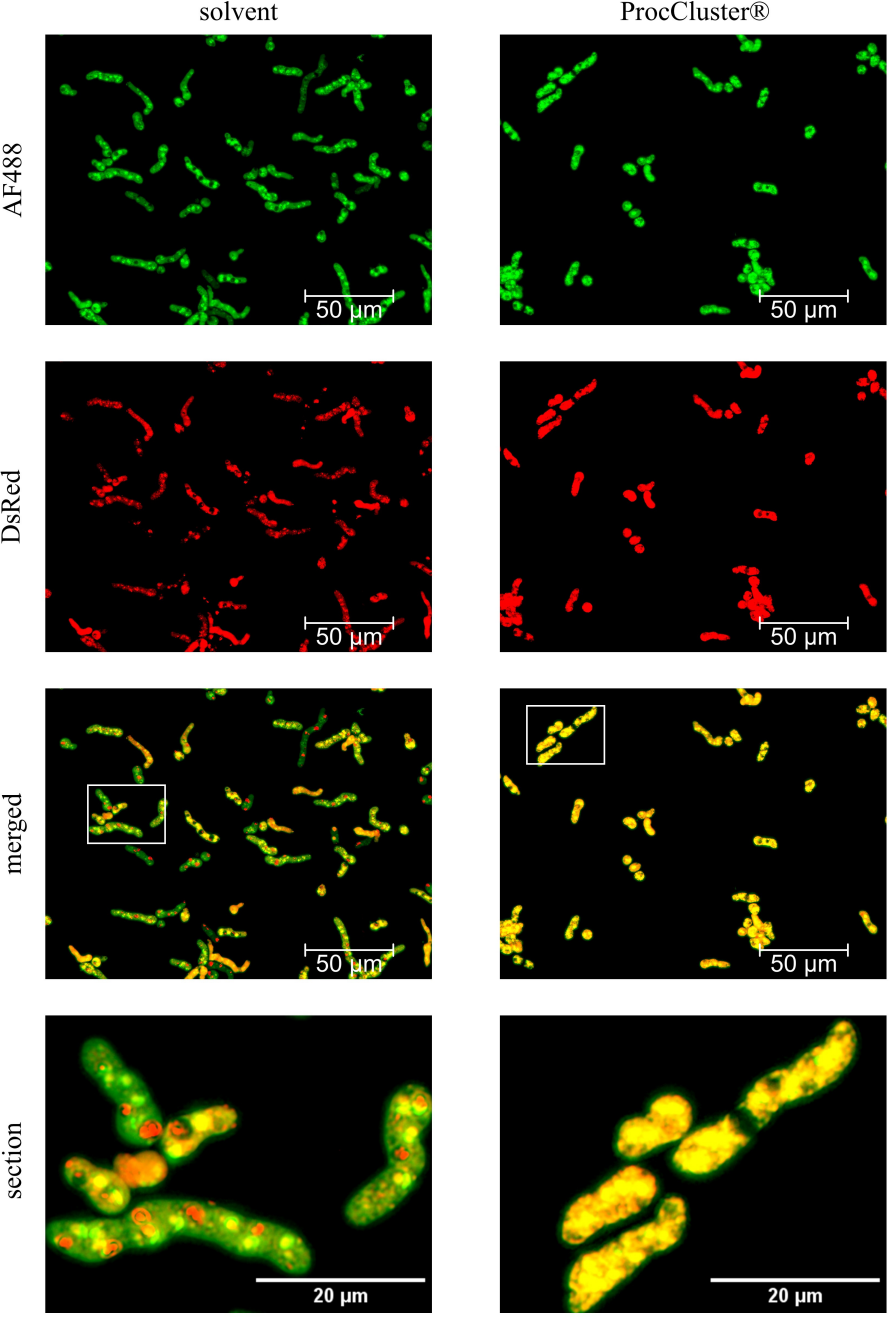
ProcCluster® treatment reduces the metabolic activity of *A. fumigatus* wt in a human cell-free environment. Conidia of *A. fumigatus* wt were grown for 8 h in the absence of any inhibitor. Subsequently, the fungus was treated with 5 mM ProcCluster® or solvent (H_2_O). After a further 1.5 h of incubation, *A. fumigatus* was stained with 10 µM FUN™1-solution and treated again with 5 mM ProcCluster® or solvent for another 30 min. FUN™1 diffuses as a green fluorophore through the fungal cytoplasm. In metabolically active cells, it is metabolized in vacuoles to form a red fluorescent cylindrical structure. Thus, the green fluorescent signal in the cytoplasm is reduced (solvent-treated samples). Metabolically inactive or dead cells retain the strong cytoplasmic staining, which appears yellow in the merged images (ProcCluster®-treated samples). All fluorescence images were taken using an Axio Observer.Z1 microscope (Zeiss) at 40 × magnification. Scale bars represent 50 µm (AF488, DsRed and merged) or 20 µm (section). The images show a representative example of three independent experiments.

To analyze the effect of ProcCluster® on the metabolic activity of *A. fumigatus* wt, conidia were grown for 8 h without any inhibitor. Subsequently, 5 mM ProcCluster® or solvent were added for another 1.5 h. Finally, the fungus was incubated with FUN™1 and the inhibitor for the final half hour. After 8 h of growth and an additional 2 h of ProcCluster® treatment, most conidia germinated and formed hyphae (**Figure 2**). As shown in **Figure 1C**, the growth of *A. fumigatus* wt was reduced as a result of ProcCluster® treatment compared to the solvent-treated control. In the absence of the inhibitor, FUN™1 creates vacuoles and is condensed to red fluorescent CIVS in most of the indicated fungi. In the same fungi, the fluorescent intensity of the dye in the cytoplasm appears to be reduced compared to that in fungi that did not produce any CIVS (**Figure 2** left panel). Therefore, it can be assumed that the majority of the solvent-treated fungi are viable and metabolically active. In contrast, treatment with ProcCluster® completely inhibited the formation of CIVS. Although vacuoles are visible in some fungi, they appear to be completely empty. At the same time, the cytoplasm of all hyphae glows in a bright green-yellow color (**Figure 2** right panel).

In contrast to the solvent, the presence of ProcCluster® efficiently reduced the viability of *A. fumigatus*.

### 3.3 The antifungal effect of ProcCluster® is reversed by the addition of CaCl_2_

**Figure 3:**
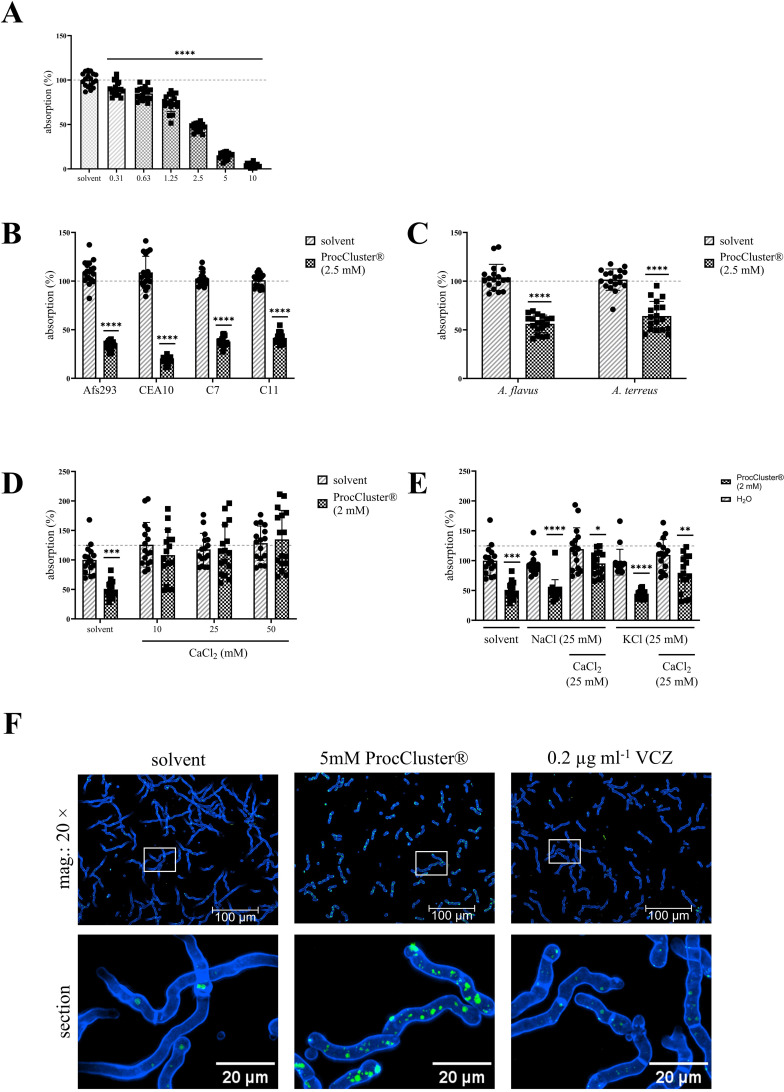
The addition of CaCl_2_ reverses the antifungal effect of ProcCluster® in a human cell-free environment. *A*. *fumigatus* **(A, D, E)**, as well as the *A*. *fumigatus* strains (Af293, CEA10 and the azole-resistant isolates C7 and C11) **(B)**, and other *Aspergillus* species (*A. flavus* and *A*. *terreus*) **(C)** were treated with the indicated concentrations of ProcCluster® or solvent (H_2_O) in a 96-well plate. After 48 h of incubation, absorption was measured at 620 nm. The absorption of the fungi growing in media without inhibitor or solvent was arbitrarily set to 100% (absorption in %). **(D, E)** To identify whether ProcCluster® inhibits the growth of *A*. *fumigatus* by influencing the ion flux in the fungi, conidia were additionally treated with different concentrations of CaCl_2_ **(D)** or 25 mM NaCl and KCl with or without 25 mM CaCl_2_ **(E)**. Finally, *A*. *fumigatus* wt was stained for intracellular Ca^2+^. For this purpose, conidia were grown for 8 h and then treated with 5 mM ProcCluster®, solvent or 0.2 µg ml^-1^ VCZ for 1 h. Fungi were stained with Calbryte™ 520 AM (green) and calcofluor white (blue) and additionally treated with the tested substances for another hour. All fluorescent images were taken using an Axio Observer.Z1 microscope (Zeiss) at 20 × magnification. Scale bars represent 100 µm (mag.: 20 ×) and 20 µm (image section). Images show one representative example of three independent experiments **(F)**. **(A–E)** Diagrams show the mean (± SD) of the results from three **(A–C)** or four **(D, E)** independent experiments including 6 **(A–C)** or 4 **(D, E)** technical replicates. Statistical significance was assessed by one-way ANOVA followed by Dunnett’s multiple comparison, comparing the mean of all samples to the solvent-treated control **(A)** or by a one sample t-test between the inhibitor and solvent-treated samples of each *A*. *fumigatus* strain **(B)** or *Aspergillus* species **(C)** or between ProcCluster®- and solvent-treated fungi **(E)** (****p ≤ 0.0001, ***p ≤ 0.001, **p ≤ 0.01, *p ≤ 0.05). mag., magnification.

Our previous results show that ProcCluster® is an efficient antifungal agent against *A. fumigatus*. So far, the inhibitory effect has been analyzed after a maximum of 10 h. To investigate the antifungal effect over longer periods, conidia of *A. fumigatus* were seeded in 96-well plates and incubated with different concentrations of ProcCluster® (0.31 mM–10 mM) or solvent for 48 h. During this time, *A. fumigatus* forms a thick layer of mycelium that absorbs high amounts of light at 620 nm. The absorption correlated with the density of the mycelium. Notably, the absorption rate decreased with increasing concentrations of ProcCluster®. Although the inhibitory effect achieved after 48 h was weaker than that after 10 h (**Figure 1A**), 2.5 mM of the substance still reduced the growth of the fungus by 50% (**Figure 3A**). Procaine HCl excerted a similar effect under the same conditions (**Supplementary Figure 2**).

To demonstrate a broad antifungal activity of ProcCluster®, its effect on the *A. fumigatus* strains Af293 and CEA10, as well as the azole-resistant isolates C7-NRZ-2016-121 (C7) and C11-NRZ-20147-040 (C11), were examined. The lower sensitivity of C7 and C11 to VCZ compared to the other tested *A. fumigatus* strains was confirmed (**Supplementary Figure 3**). Of particular note is the sensitivity of strain Af293 to VCZ, which shows a pattern similar to that of the azole-resistant strain C11. For both strains, fungal growth was reduced by only half after treatment with 0.1 µg ml^-1^ VCZ. In contrast, the same concentration had a much stronger inhibitory effect on the *A. fumigatus* strain, which was used in most of the experiments presented in this study, and on the CEA10 strain. However, regardless of azole sensitivity, 48 h of treatment with 2.5 mM ProcCluster® reduced the absorption rate of the mycelial layer of all *A. fumigatus* strains tested (**Figure 3B**). Although *A. fumigatus* is by far the most common species in invasive aspergillosis, other species, such as *A. flavus* and *A. terreus*, also have the potential to infect the human lungs. Therefore, growth inhibition by ProcCluster® was also observed for these species. Compared to the solvent-treated control, ProcCluster® reduced the growth of *A. flavus* and *A. terreus* to almost 50% (**Figure 3C**).

It has been indicated that LAs act antimycotic by interfering with the fungal Ca^2+^ homeostasis. Thus, *A. fumigatus* was treated with 2 mM ProcCluster® and the indicated concentrations of CaCl_2_, NaCl and/or KCl. Absorption measurements revealed that the addition of CaCl_2_ completely reversed the effect of ProcCluster®. After 48 h of incubation, ProcCluster® alone inhibited fungal growth as expected. In the presence of CaCl_2_ the inhibitor-treated fungi grew as fast as the solvent-treated ones (**Figure 3D**). In contrast, supplementation with NaCl or KCl alone did not affect the growth-inhibiting effect of ProcCluster®. However, when one of the salts was added to *A. fumigatus* along with CaCl_2_, the growth of the fungus was again unaffected or less affected by the tested inhibitor (**Figure 3E**). To study changes in intracellular Ca^2+^ levels during treatment with ProcCluster® in more detail, *A. fumigatus* was grown for 8 h and then treated with 5 mM ProcCluster®, solvent or 0.2 µg ml^-1^ VCZ for 1 h. Afterwards, fungi were stained with Calbryte™ 520 AM (green) and calcofluor white (blue) in the presence of the tested substances for an additional hour. After two washing steps with CaCl_2_-supplemented media, intracellular Ca^2+^ levels were analyzed by fluorescence microscope. The images show intensely green stained structures in the ProCluster®-treated fungi, indicating Ca^2+^ accumulation in the presence of this substance. In comparison, the treatment with solvent or VCZ alone resulted in mild green dyed structures that were present in smaller number (**Figure 3F**).

In summary, our results suggest that ProcCluster® inhibits the growth of *A. fumigatus* by interfering with fungal Ca^2+^ homeostasis.

### 3.4 ProcCluster® and procaine HCl lower viral titers and fungal growth in coinfection with IAV and *A. fumigatus in vitro*

**Figure 4:**
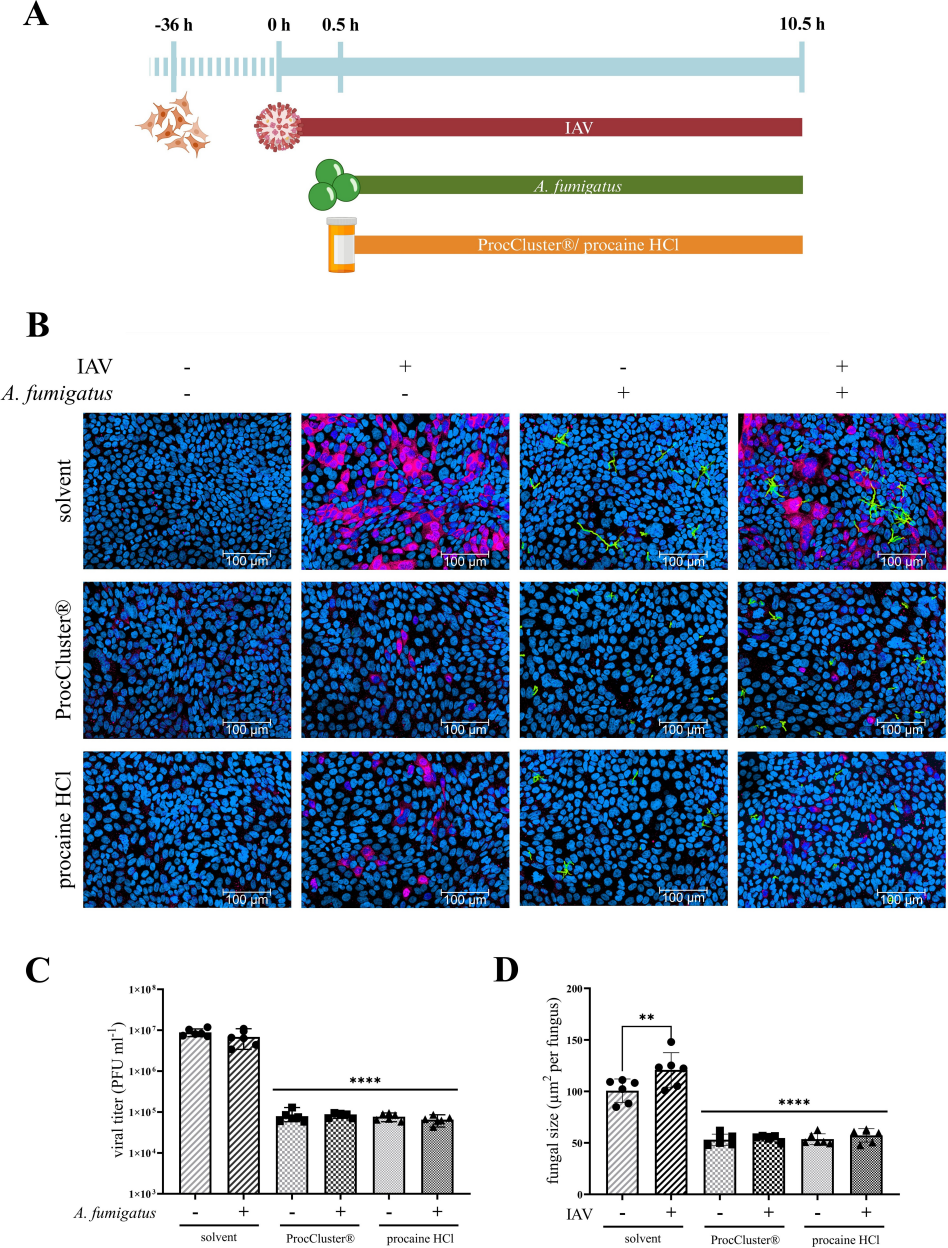
ProcCluster® and procaine HCl have an antimicrobial effect on *A*. *fumigatus* and IAV during coinfection on Calu-3 cells. Calu-3 cells were infected with IAV (H1N1; 0.5 multiplicity of infection (MOI)) for 30 min or left uninfected and were subsequently infected with conidia of *A*. *fumigatus* (10 MOI) for further 10 h or left uninfected again. Concurrent with fungal infection, cells were treated with 2.5 mM ProcCluster®, procaine HCl or solvent (H_2_O). **(A)** Schematic representation of the experimental setup. **(B)** Immunofluorescence images showing IAV-infected cells in red (stained with mouse anti-IAV-NP antibody and AlexaFluor674-conjugated donkey anti-mouse antibody), Hoechst 33342-stained nuclei of Calu-3 cells in blue, and GFP-expressing *A*. *fumigatus* in green. All images were taken with an Axio Observer.Z1 microscope (Zeiss) at 20 × magnification. Scale bars represent 100 µm. The images show one representative example from three independent experiments. **(C)** Viral titers were analyzed using standard plaque assay. The graph shows the viral titer as plaque forming unit (PFU) ml^-1^. **(D)** Immunofluorescence images were also used to quantify the growth (µm^2^ per fungus) of *A*. *fumigatus* in the absence and presence of the tested drugs. **(C, D)** Graphs show the mean (± SD) of the results of three independent experiments, including duplicates. Statistical significance was assessed by one-way ANOVA followed by Tukey’s multiple comparison test. Statistical significance refers to the comparison between ProcCluster® or procaine HCl-treated samples and the corresponding solvent-treated control (****p ≤ 0.0001, **p ≤ 0.01).

Since our own results recently demonstrated antiviral activity of the two procaine prodrugs ProcCluster® and procaine HCl, their effects on IAV and *A. fumigatus in vitro* coinfections were of interest. To study this issue, Calu-3 cells (**Figure 4**), A549 cells (**Supplementary Figure 4**) or HBEpCs (**Supplementary Figure 5**) were infected with IAV, using a MOI of 0.5 (Calu-3 cells, A549 cells) or 5 (HBEpCs) for 30 min. Subsequently, the cells were treated with 2.5 mM of the tested drugs or solvent. At the same time, conidia of *A. fumigatus* (10 MOI) were added for the secondary infection and samples were incubated for further 10 h (**Figure 4A**).

First, immunofluorescence assays were performed by staining the nuclei of the host cells with Hoechst 33342 (blue) and the NP of IAV with a mouse anti-NP antibody and a donkey anti-mouse IgG Alexa Fluor™ 647 (red). GFP-expressing *A. fumigatus* is visible in green. During solvent treatment, more than half of the host cells were infected with IAV for both single- and coinfection. Furthermore, most conidia of *A. fumigatus* formed hyphae after 10 h of solvent treatment. In the presence of ProcCluster® and procaine HCl, the number of IAV-infected cells and the hyphal size of *A. fumigatus* was decreased significantly (**Figure 4B**; **Supplementary Figures 4A**, **5A**). Further investigations revealed results to those obtained using immunofluorescence microscopy. Standard plaque assays were performed to determine the progeny viral titers of IAV in the presence or absence of the tested drugs. In particular, in Calu-3 cells, treatment with 2.5 mM ProcCluster® and procaine HCl resulted in a 99% reduction in virus titers, regardless of the presence of the fungus (**Figure 4C**). Comparable tests in A549 cells and HBEpCs revealed a smaller but significant reduction in IAV titers (**Supplementary Figures 4B**, **5B**). Immunofluorescence assays were also used to quantify fungal growth. In Calu-3 cells, both drugs were able to reduce the growth of *A. fumigatus* to less than half of that of the solvent-treated fungi (**Figure 4D**). Similar results were observed in experiments performed in A549 cells or HBEpCs (**Supplementary Figures 4C**, **5D**). As expected, in the solvent-treated control and under coinfective conditions, the growth of *A. fumigatus* was higher that during a single fungal infection. However, after treatment with an inhibitor, these differences disappeared (**Figure 4D**; **Supplementary Figures 4C**, **5C**).

**Figure 5:**
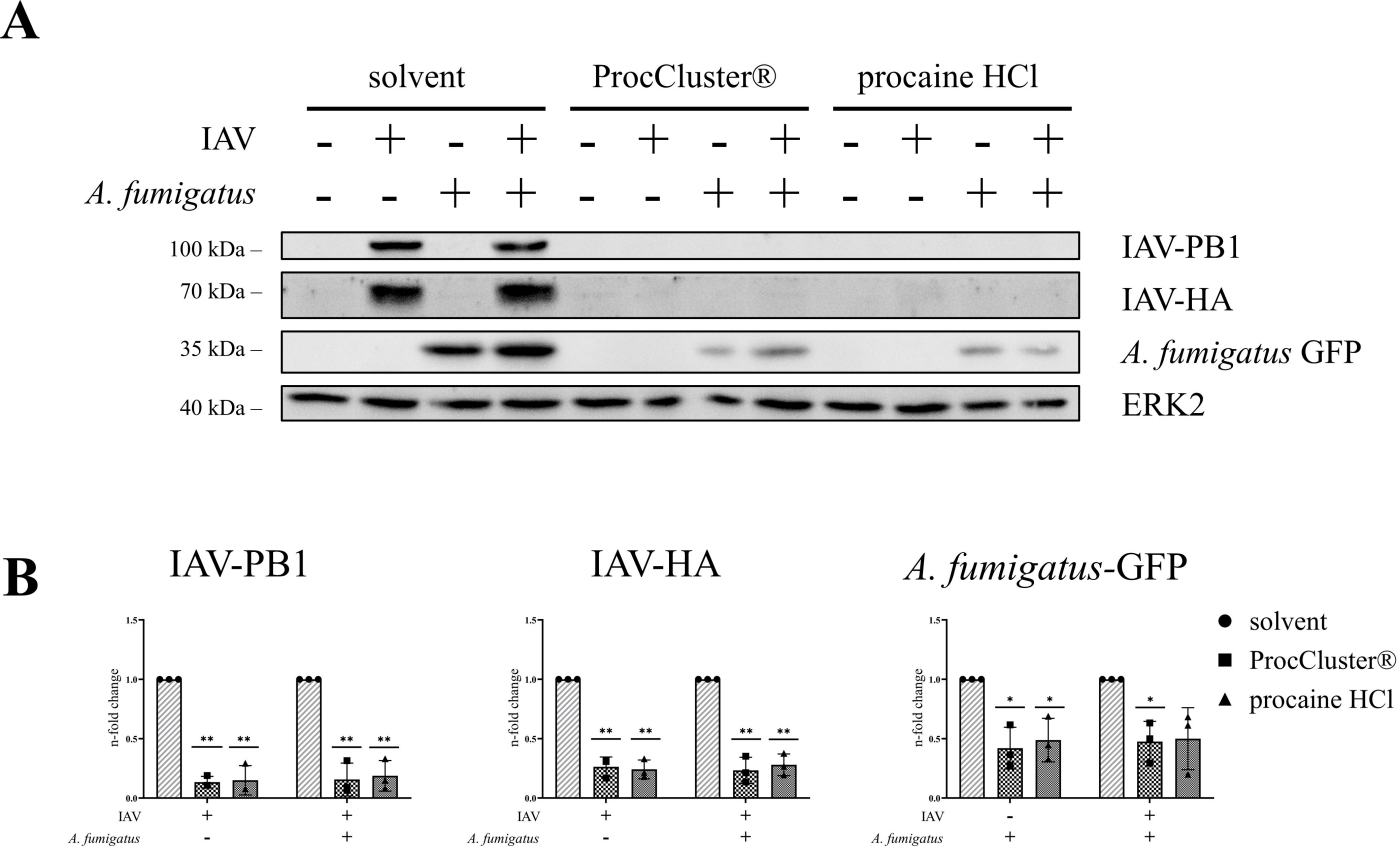
IAV protein expression levels are reduced in the presence of ProcCluster® and procaine HCl during coinfection of Calu-3 cells. Calu-3 cells were infected with IAV (H1N1; 0.5 MOI) for 30 min or left uninfected and were subsequently infected with conidia of *A*. *fumigatus* (10 MOI) for further 10 h or left uninfected. Concurrent with fungal infection, cells were treated with 2.5 mM ProcCluster®, procaine HCl or solvent (H_2_O). The antimicrobial effects of the drugs on the level of protein expression of IAV-PB1, IAV-HA, and *A*. *fumigatus*-GFP were examined by Western blot analysis. **(A)** Western blot images represent one example out of three independent experiments. **(B)** Western blot images were also used to quantify the levels of the aforementioned proteins. Protein signals were analyzed and normalized to the ERK2 signal. The solvent-treated single infection was arbitrarily set to one. Diagrams show the mean (± SD) of three independent experiments. Statistical significance was assessed by one sample t-test. Significances relate to the comparison between ProcCluster® or procaine HCl-treated samples and the appropriate solvent-treated control (**p ≤ 0.01, *p ≤ 0.05).

To analyze the effect of ProcCluster® and procaine HCl on viral protein expression, Western blot assays were performed after 10 h of coinfection (**Figure 5A**). As a result of inhibitor treatment, protein synthesis of IAV-PB1 and IAV-HA was significantly reduced. In line with the fungal size, GFP expression of *A. fumigatus* decreased during treatment with the tested drugs (**Figure 5B**).

These data indicate that ProcCluster® and procaine HCl can reduce viral replication of IAV and simultaneously reduce the growth of *A. fumigatus* during coinfection with both pathogens *in vitro*.

### 3.5 Combination treatment with ProcCluster® and favipiravir enhances the inhibition of IAV replication during *in vitro* coinfection

**Figure 6:**
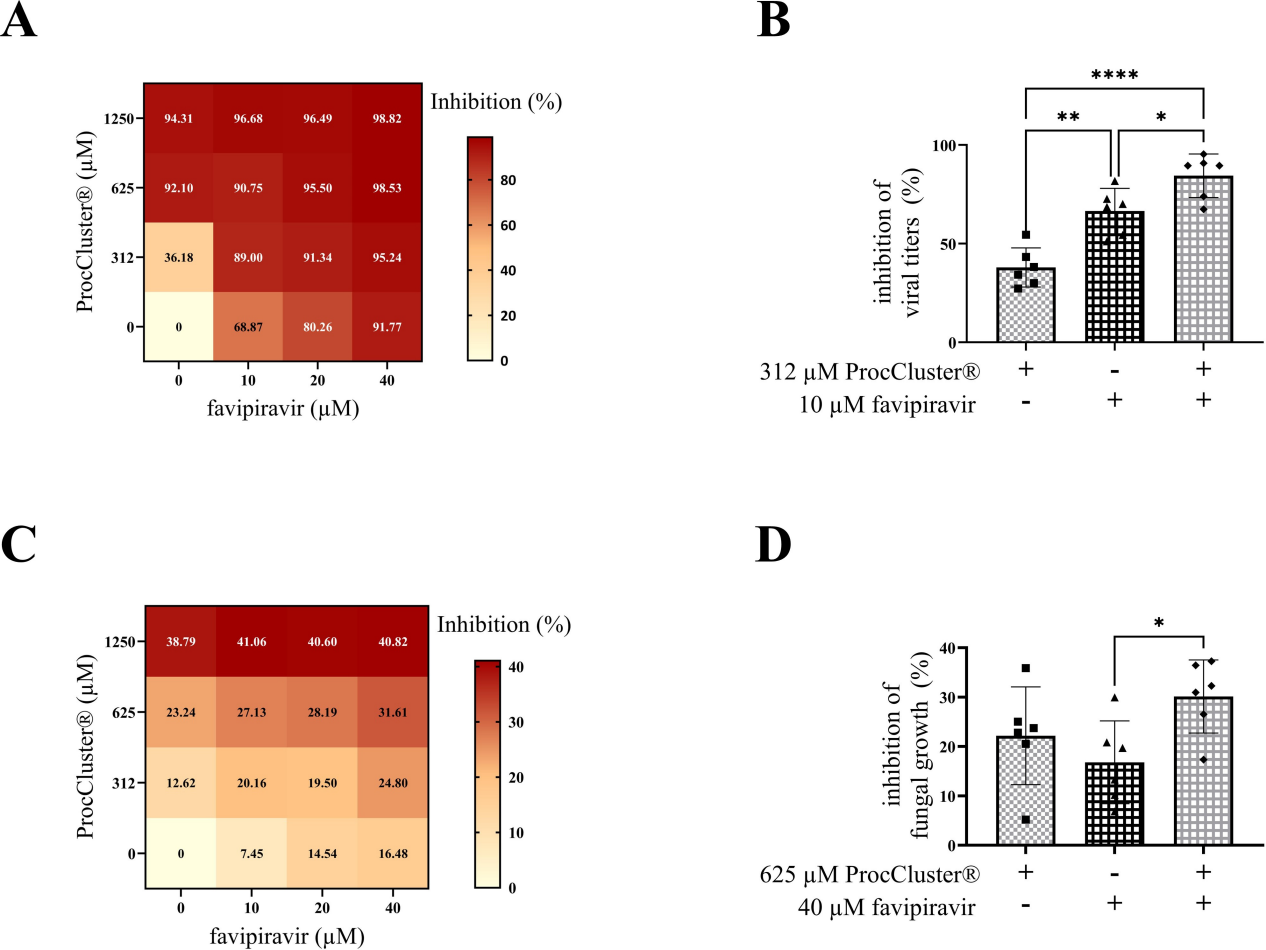
Combination treatment with ProcCluster® and favipiravir enhanced the inhibitory effect on IAV titers during coinfection on Calu-3 cells. Calu-3 cells were infected with IAV (H1N1; 0.5 MOI) for 30 min and subsequently infected with conidia of *A*. *fumigatus* (10 MOI) for further 10 h. Concurrent with fungal infection, cells were treated with different concentrations of ProcCluster® and/or favipiravir or with the indicated solvent (H_2_O and/or DMSO). The inhibitory effect of combination treatment with both drugs on IAV titers was analyzed by plaque assay **(A, B)** and fungal growth was analyzed by fluorescence microscopy **(C, D)**. **(A, C)** Results were plotted as heatmaps, showing the mean inhibitory effect in %. **(B, D)** Graphs showing the inhibitory effects of 312 µM ProcCluster® and 10 µM favipiravir on viral propagation **(B)** and the inhibitory effects of 625 µM ProcCluster® and 40 µM favipiravir on the growth of *A*. *fumigatus* **(D)**. Diagrams show the mean (± SD) of the results of three independent experiments, including duplicates. Statistical significance was evaluated by one-way ANOVA followed by Tukey’s multiple comparison test (****p ≤ 0.0001, **p ≤ 0.01, *p ≤ 0.05).

Treatment with two or more medications is an alternative strategy for improving classic antiviral therapy. Here, we combined different concentrations of ProcCluster®, a substance with antiviral and antifungal properties, and favipiravir, an antiviral drug that inhibits viral polymerase activity, during 10 h of coinfection with IAV (0.5 MOI) and *A. fumigatus* (10 MOI). Determination of progeny IAV titers by standard plaque assay revealed that both drugs alone are already very effective antivirals (**Figure 6A**). In particular 10 µM favipiravir already inhibited almost 70% of the solvent-treated virus titers. The combination of the lowest concentration of ProcCluster® (312 µM) and favipiravir (10 µM) significantly increased the inhibitory effect, compared to the monotreatment with one of the tested substances (**Figure 6B**). However, the higher the concentration of one or both drugs, the smaller the differences between combination and monotherapy become (**Figure 6A**). Nevertheless, using the online tool SynergyFinder 3.0, the results indicate an additive antiviral effect for the combination of ProcCluster® and favipiravir, during coinfection with IAV and *A. fumigatus in vitro* (**Supplementary Figure 6A**).

In addition, the effects of combination treatment with both drugs was also tested for the growth of *A. fumigatus* during coinfections. Again, fluorescence microscopy was used to determine fungal size. Surprisingly, treatment with favipiravir alone already had a small impact on the inhibition of fungal growth (**Figure 6C**). In the presence of ProcCluster®, the inhibitory effect was even increasing. However, the combination of 625 µM ProcCluster® and 40 µM favipiravir resulted in minor differences compared to the treatment with either of the tested drugs (**Figure 6D**). Although the combination of the two substances inhibited fungal growth to a lesser extent than the inhibition rates observed for IAV (**Figures 6A, B**), the synergy test still resulted in a slight additive effect (**Supplementary Figure 6B**).

Taken together, the combinatory treatment ProcCluster® and favipiravir resulted in an enhanced inhibitory effect, particularly against IAV replication during IAV and *A. fumigatus* coinfection *in vitro*. In addition, much lower concentrations of each drug are required to achieve high antiviral activity.

## 4 Discussion

IPA remains a life-threatening disease in high-risk patients and the number of cases is continuously increasing every year (Zilberberg et al., 2019). In addition to classical risk factors, such as neutropenia and hematologic malignancies (Ledoux and Herbrecht, 2023), other predispositions, such as severe viral lung infections, have also been identified as indicators of aspergillosis (van de Veerdonk et al., 2017; Schauwvlieghe et al., 2018). Coinfections with influenza viruses and *Aspergillus* species are known to affect both, immunocompromised as well as immunocompetent patients, and are associated with high mortality rates (van de Veerdonk et al., 2017; Schauwvlieghe et al., 2018). To date, there are no universal therapeutic approaches for treating IAPA. Although combating each pathogen individually seems obvious, one should consider how the antiviral or antifungal drug affects the other pathogen. Although murine models have shown that treatment of viral infections with oseltamivir or baloxavir marboxil reduces the fungal burden of *A. fumigatus* (Seldeslachts et al., 2021a, 2021), the effect of antifungals on viral infections is not fully understood. Interestingly, itraconazole, a drug of the antifungal triazole family, has been reported to inhibit influenza virus infection (Schloer et al., 2019). In contrast, amphotericin B, a polyene antifungal agent, appears to promote influenza virus replication by inhibiting interferon-induced transmembrane protein 3 (IFTIM3) in host cells (Roethl et al., 2011; Lin et al., 2013). An inhibitor that combines both antiviral and antifungal properties could overcome the risk of promoting the propagation of one pathogen while combating the other. Therefore, the search for drugs that target pathogen-supporting cellular functions of virus-infected human cells and fungal factors could be an alternative strategy. For example, aspirin, a nonsteroidal anti-inflammatory drug (NSAIDs), inhibits IAV propagation by downregulating the IκB kinase (IKK)/nuclear factor-κB (NF-κB) signaling pathways in host cells (Mazur et al., 2007). As an antifungal agent, aspirin inhibits biofilm formation of *Candida* species (Al-Bakri et al., 2009; Chan et al., 2021). Ibuprofen, another NSAID, also has antimicrobial properties that have been demonstrated against *Aspergillus* species (Obad et al., 2015). Furthermore, LAs such as lidocaine, bubivacaine, and procaine affect the growth of various fungi, including *Candida* and *Aspergillus* species (Rodrigues et al., 2000, 2006). In addition, LAs have been reported to have antiviral effects against herpes simplex viruses, influenza viruses and West-Nile viruses (Fuchs and Levanon, 1978; Bastos et al., 2020; Häring et al., 2021; Liu et al., 2023; Häring et al., 2024). However, none of these drugs have been tested during coinfection with IAV and *A. fumigatus*.

Here, we investigated the antifungal properties of the LA procaine prodrugs ProcCluster® and procaine HCl against *A. fumigatus*. Since we recently demonstrated antiviral activity against SARS-CoV-2 and influenza viruses for both drugs (Häring et al., 2021, 2023, 2024), we further analyzed whether these two drugs are effective against both pathogens during IAV and *A. fumigatus* coinfection *in vitro*.

Our results indicate that ProcCluster® and procaine HCl inhibit the growth of *A. fumigatus* after 10 h of treatment (**Figures 1A, B**; **Supplementary Figure 1**). In fact, ProcCluster® still reduced fungal growth, when added 8 h after incubation (**Figures 1C, D**). At that time, *A. fumigatus* had already developed short hyphae (Kwon-Chung and Sugui, 2013). Hyphal growth and lung invasion are hallmarks of IPA, and antifungal therapy is usually initiated at this growth stage (Latge and Chamilos, 2019). Thus, targeting germinating fungi should be an essential property of all antifungal drugs. Since hyphal growth is accelerated by direct interaction of IAV with the hyphae of *A. fumigatus* (König et al., 2023), we analyzed how ProcCluster® affects fungal growth during incubation with IAV particles. Notably, in the presence of ProcCluster® the virus-induced hyphal growth was reduced to a similar level as seen for the unstimulated and inhibitor-treated control (**Figures 1E, F**). To visualize ProcCluster®-mediated effects on the metabolic activity of the fungus, FUN™1 staining was performed. The red fluorescent CIVS, indicating metabolic action, are visible after only 1 h of staining. As expected, treatment with solvent did not affect fungal viability, whereas the addition of ProcCluster® mainly resulted in metabolically inactive fungi (**Figure 2**).

Since our results point to an antifungal property of ProcCluster® against *A. fumigatus*, its broad efficacy should be tested. Thus, the hyphal growth of other *A. fumigatus* strains, including azole-resistant strains, as well as other *Aspergillus* species was examined upon ProcCluster® treatment. The azole-resistant strains C7 and C11 are resistant against VCZ, itraconazole and posaconazole. Resistance of C7 is based on a mutation (TR_34_/L98H) in the *cyp51a* gene, which is the most common cause of azole-resistance (Barber et al., 2021). In European hospitals, azole-resistant *A. fumigatus* strains are an emerging problem (van der Linden et al., 2011; Lestrade et al., 2019) and are thought to be related to the widespread use of azole fungicides in the environment (van der Linden et al., 2015). Because triazoles are recommended as first-line treatment, patients infected with azole-resistant *A. fumigatus* strains have a worse disease outcome than those infected with an azole-susceptible strain (van der Linden et al., 2011; Lestrade et al., 2019). As a second-line treatment, amphotericin B is recommended. However, the polyene is related to severe side effects, especially nephrotoxicity, and the growing number of amphotericin B-resistant strains is concerning (Laniado-Laborin and Cabrales-Vargas, 2009). Therefore, the search for new antimycotics that are effective against resistant isolates remains relevant. The *A. fumigatus* strains tested in this study were all susceptible to ProcCluster® (**Figures 3A, B**), regardless of their azole susceptibility (**Supplementary Figure 3**). Although *A. fumigatus* causes the majority of invasive aspergillosis worldwide, *A. flavus* is the predominant cause of aspergillosis in Asian and Middle Eastern countries and can invade the lungs, the paranasal sinus and the central nervous system (Gregg and Kauffman, 2015; Rudramurthy et al., 2019). Infections with other *Aspergillus* species like *A. terreus* are rare but are associated with a poor course of disease (Lass-Florl et al., 2005; Lass-Flörl et al., 2021). Furthermore, *A. flavus* and *A. terreus* are intrinsically resistant to polyenes such as amphotericin B (Lass-Florl et al., 2005; Rudramurthy et al., 2019). Thus, triazoles like VCZ remain the main treatment option for infections caused by *A. flavus* or *A. terreus*. Moreover, triazoles are also associated with severe side effects and alternative antifungal drugs are required (Wang et al., 2010; Chai et al., 2022). Although *A. flavus* and *A. terreus* seem to be less sensitive to ProcCluster® than the *A. fumigatus* strains tested, growth was reduced to almost 50% after 48 h of treatment (**Figure 3C**).

Few publications have indicated that LAs act as antifungal agents by interfering with fungal Ca^2+^ homeostasis (Rodrigues et al., 2000, 2006). Our data point to a similar assumption. By adding high concentrations of CaCl_2_ to ProcCluster®-treated *A. fumigatus*, we were able to reverse the growth-inhibiting effect of the test substance (**Figure 3D**). In contrast, the addition of similar amounts of NaCl and KCl did not result in any changes (**Figure 3E**). Furthermore, intracellular staining revealed increased Ca^2+^ accumulation in *A. fumigatus* hyphae in the presence of ProcCluster® (**Figure 3F**). Ca^2+^ is an essential second messenger, not only in animal but also in fungal cells. Cytosolic Ca^2+^ concentrations are highly regulated and molecules involved in maintaining intracellular Ca^2+^ homeostasis in *Aspergillus* species are also suggested to regulate hyphal growth and the pathogenicity of the fungi (Cramer et al., 2008; de Castro et al., 2014; Li et al., 2024). The inhibition of these proteins represents a promising mechanism for the development of new antifungal drugs (Fox and Heitman, 2002; Pinchai et al., 2009). As intracellular Ca^2+^ ions are stored in organelles such as vacuoles, the endoplasmic reticulum and mitochondria (Tisi et al., 2016), our results indicate that ProcCluster® inhibits the Ca^2+^ release into the cytoplasm, probably by blocking Ca^2+^ channels, and by that interrupting Ca^2+^-dependent signaling pathways. As consequence, high extracellular Ca^2+^ concentrations can compensate for the lack of cytosolic Ca^2+^, thereby counteracting the antifungal properties of ProcCluster®. LAs are reported to act on different cellular targets in humans. Thus, they exhibit multiple properties, such as preventing or relieving pain, as well as anti-inflammatory or antitumor effects (Cassuto et al., 2006; Li et al., 2018, 2021). Procaine targets the MAPK pathway or DNA methylation in human cells (Li et al., 2018, 2021). Furthermore, our own studies have shown that ProcCluster® affects different parts of viral replication, probably by interacting with different cellular elements (Häring et al., 2023). Factors such as MAPKs are highly conserved in *A. fumigatus* and are involved in biofilm formation, stress tolerance, and virulence (Hagiwara et al., 2014; Manfiolli et al., 2018; Day and Quinn, 2019). In addition to the interference of fungal Ca^2+^ homeostasis, ProcCluster® may affect fungal growth by other mechanisms.

As mentioned, ProcCluster® and procaine HCl inhibit IAV replication *in vitro* (Häring et al., 2024). Because IAV and *A. fumigatus* infection leads to severe disease progression and treatment options are rare, the test substances were examined for their antimicrobial effects during IAV and *A. fumigatus* coinfection *in vitro*. Treatment with either LA efficiently reduced the viral titers of IAV and lowered the growth of *A. fumigatus* (**Figures 4C, D**, **5**, **Supplementary Figures 4**, **5**). Nevertheless, the treatment with antiviral drugs is still the first choice and the development of novel antiviral agents remains essential (Shin and Seong, 2019). Instead of replacing classical therapeutics, combining them with host-directed therapies could represent a promising option in clinics. Combination therapies for the treatment of influenza virus infections have synergistic or additive effects by acting on different parts of the replication cycle, reduce drug dosage and consequently drug toxicity and may reduce the risk of developing drug resistances (Koszalka et al., 2022; Batool et al., 2023). Therefore, we combined different concentrations of ProcCluster® and favipiravir to treat coinfected Calu-3 cells. Favipiravir is a broad-spectrum antiviral drug that inhibits the RNA-dependent polymerase of various RNA viruses, including influenza viruses, coronaviruses and ebolaviruses (Furuta et al., 2005; Nagata et al., 2015; Joshi et al., 2021). Compared to other antivirals, such as oseltamivir, favipiravir appears less vulnerable to the development of resistant influenza virus strains (Shiraki and Daikoku, 2020). As potent antiviral drugs, treatment with ProcCluster® and favipiravir alone resulted in high inhibition rates (**Figure 6A**). However, especially at lower concentrations, the combination of both drugs led to significantly stronger antiviral effects (**Figures 6A, B**). Thus, the combination of ProcCluster® and favipiravir has the potential to reduce the concentration of each drug by maintaining high antiviral property. Regarding fungal growth, combination therapy with the same drugs had only slight additive effects (**Figures 6C, D**). However, it was surprising to find that favipiravir alone reduced fungal growth. Similar results were reported when coinfected mice were treated with oseltamivir or baloxavir marboxil (Seldeslachts et al., 2021a, 2021). The authors explained these effects by the reduced number of alveolar macrophages in untreated, virus-infected mice but not in antiviral-treated mice. By reducing viral burden, the immune system can remain stable to fight secondary fungal infection (Seldeslachts et al., 2021a, 2021). However, our experimental setup did not contain any immune cells. As we have shown that the interaction of IAV with *A. fumigatus* results in enhanced hyphal growth (König et al., 2024), favipiravir probably inhibited this effect by blocking viral propagation, thereby reducing fungal growth.

It has already been discussed that the concentrations of ProcCluster® and procaine HCl used here exceed the tolerable serum concentrations of procaine (Häring et al., 2023). However, there are no publications on the administration levels of ProcCluster® in patients and using ProcCluster® along with other drugs could downregulate the required concentrations. For cell culture experiments, the nontoxic concentrations of ProcCluster® and procaine HCl were recently determined (Häring et al., 2023, 2024). Nonetheless, since this study was conducted under cell culture conditions, systemic factors, such as the involvement of the immune system or the surrounding tissue, are missing. Thus, further experiments and additional models, such as animal experiments, are needed to determine the clinical potential of ProcCluster® and procaine HCl as antimicrobial agents against viral and fungal infections.

Overall, ProcCluster® and procaine HCl inhibited growth of *A. fumigatus* and other *Aspergillus* species in a human cell-free environment. Even in *in vitro*-coinfection scenarios with IAV and *A. fumigatus*, both drugs effectively reduced viral and fungal propagation. In the future, the combination of ProcCluster® with classic antivirals or antifungals could reduce the effective dose of the drugs by maintaining high antimicrobial activity.

## Data Availability

The raw data supporting the conclusions of this article will be made available by the authors, without undue reservation.

## Supplementary material

Supplementary Figure 1 In the presence of procaine HCl, the growth of *A. fumigatus* is efficiently inhibited in a human cell-free environment. Conidia of *A. fumigatus* were incubated with the indicated concentrations of procaine HCl, solvent (H_2_O) or 0.2 µg ml^-1^ VCZ for 10 h and growth of the fungus was quantified by fluorescence microscopy. **(A)** Quantification is given as µm^2^ per fungus. The diagram shows the mean (± SD) of the results of three independent experiments, including duplicates. **(B)** Selected fluorescence images showing GFP-expressing fungi treated with solvent (H_2_O), 0.2 µg ml^-1^ VCZ, 1.25 mM or 2.5 mM procaine HCl. Fluorescence images show one representative example of three independent experiments. **(A)** Statistical significance was assessed by one-way ANOVA followed by Dunnett’s multiple comparison test, comparing the mean of all samples with the solvent-treated sample (*****p* ≤ 0.0001, ***p* ≤ 0.01, **p* ≤ 0.05).

Supplementary Figure 2 Procaine HCl treatment affects the growth of *A. fumigatus* after 48 h. Conidia of *A. fumigatus* were treated with different concentrations of procaine HCl in a 96-well plate. After 48 h of incubation, the absorption of the cultures was measured at 620 nm. The graph shows the absorption in %, while the absorption of *A. fumigatus* growing in medium without drug or solvent was set to 100%. Diagram shows the mean (± SD) of the results from three independent experiments including 6 technical replicates. Statistical significance was assessed by one-way ANOVA followed by Dunnett’s multiple comparison test, comparing the mean of all samples with the solvent-treated sample (A) (*****p* ≤ 0.0001, ***p* ≤ 0.01).

Supplementary Figure 3
*A. fumigatus* strain C7 is less sensitive to voriconazole treatment than other strains tested. Conidia of *A. fumigatus* (the strain used for most of the experiments) **(A)** and the *A. fumigatus* strains: CEA10 **(B)**, Af293 **(C)**, C7 **(D)** and C11 **(E)** were treated with different concentrations of VCZ or solvent (DMSO) in a 96-well plate. After 48 h of incubation, the absorbtion of the cultures was measured at 620 nm. Graphs show the absorption in %, while the absorption of *A. fumigatus* growing in medium without drug or solvent was set to 100%. Diagrams show the mean (± SD) of three independent experiments including 6 technical replicates. Statistical significance was assessed by one-way ANOVA followed by Dunnett’s multiple comparison test, comparing the mean of all samples with the solvent-treated sample **(A)** (*****p* ≤ 0.0001, ****p* ≤ 0.001).

Supplementary Figure 4 ProcCluster® and procaine HCl have an antimicrobial effect on *A. fumigatus* and IAV during coinfection on A549 cells. **(A–C)** A549 cells were infected with IAV (H1N1; 0.5 MOI) for 30 min or left uninfected and were subsequently infected with conidia of *A. fumigatus* (10 MOI) for a further 10 h or left uninfected again. Concurrent the fungal infection, cells were treated with 2.5 mM ProcCluster®, procaine HCl or solvent (H_2_O). **(A)** Immunofluorescence images showing IAV-infected cells in red (stained with mouse anti-IAV-NP antibody and AlexaFluor674-conjugated donkey anti-mouse antibody), Hoechst 33342-stained nuclei of Calu-3 cells in blue and GFP-expressing *A. fumigatus* in green. All images were taken using an Axio Observer.Z1 microscope (Zeiss) at 20 × magnification. Scale bars represent 100 µm. The images show one representative example of three independent experiments. **(B)** Viral titers were analyzed using standard plaque assay. The graph shows the viral titer as PFU ml^-1^. **(C)** Immunofluorescence images were also used to quantify the growth (µm^2^ per fungus) of *A. fumigatus* in the presence and absence of the tested drugs. **(B, C)** Graphs show the mean (± SD) of the results of three independent experiments including duplicates. Statistical significance was assessed by one-way ANOVA followed by Tukey’s multiple comparison test. Statistical significances shown in **(B, C)** refer to the comparison between ProcCluster® or procaine HCl-treated samples and the corresponding solvent-treated control (*****p* ≤ 0.0001, **p* ≤ 0.05).

Supplementary Figure 5 ProcCluster® has an antimicrobial effect on *A. fumigatus* and IAV during coinfection on HBEpCs. HBEpCs were infected with IAV (H1N1; 5 MOI) for 30 min or left uninfected and were subsequently infected with conidia of *A. fumigatus* (10 MOI) for further 10 h or were left uninfected again. Concurrent with fungal infection, cells were treated with 2.5 mM ProcCluster® or with solvent (H_2_O). **(A)** Immunofluorescence images showing IAV-infected cells in red (stained with mouse anti-IAV-NP antibody and AlexaFluor674-conjugated donkey anti-mouse antibody), Hoechst 33342-stained nuclei of Calu-3 cells in blue and GFP-expressing *A. fumigatus* in green. All images were taken using an Axio Observer.Z1 microscope (Zeiss) at 20 × magnification. Scale bars represent 100 µm. The images show one representative example of three independent experiments. **(B)** Viral titers of IAV were analyzed using standard plaque assay. The graph shows the viral titer as PFU ml^-1^. **(C)** Immunofluorescence images were also used to quantify the growth (µm^2^ per fungus) of *A. fumigatus* in the presence and absence of the tested drugs. **(B, C)** Diagrams show the mean (± SD) of the results of three independent experiments, including duplicates. Statistical significance was evaluated by one-way ANOVA followed by Tukey’s multiple comparison test. Statistical significances shown in **(B, C)** refer to the comparison between ProcCluster® or procaine HCl-treated samples and the appropriate solvent-treated control (*****p* ≤ 0.0001, ****p* ≤ 0.001, ***p* ≤ 0.01).

Supplementary Figure 6 Combination treatment with ProcCluster® and favipiravir has slight additive inhibitory effects against IAV and *A. fumigatus* during coinfection *in vitro*. The raw data of IAV titers **(A)** and hyphal growth of *A. fumigatus* **(B)**, shown in **Figure 6** were further analyzed using SynergyFinder 3.0 with the synergy model zero interaction potency (ZIP) and are shown as 2D plots generated by SynergyFinder.

## References

[B1] Abed Y. Boivin G. (2017). A review of clinical influenza A and B infections with reduced susceptibility to both oseltamivir and zanamivir. Open Forum Infect. Dis. 4, ofx105. doi: 10.1093/ofid/ofx105 28852674 PMC5569976

[B2] Al-Bakri A. G. Othman G. Bustanji Y. (2009). The assessment of the antibacterial and antifungal activities of aspirin, EDTA and aspirin-EDTA combination and their effectiveness as antibiofilm agents. J. Appl. Microbiol. 107, 280–286. doi: 10.1111/j.1365-2672.2009.04205.x 19302313

[B3] Balajee S. A. Marr K. A. (2002). Conidial viability assay for rapid susceptibility testing of *Aspergillus* species. J. Clin. Microbiol. 40, 2741–2745. doi: 10.1128/JCM.40.8.2741-2745.2002 12149322 PMC120618

[B4] Barber A. E. Sae-Ong T. Kang K. Seelbinder B. Li J. Walther G.. (2021). *Aspergillus fumigatus* pan-genome analysis identifies genetic variants associated with human infection. Nat. Microbiol. 6, 1526–1536. doi: 10.1038/s41564-021-00993-x 34819642

[B5] Bartley P. S. Deshpande A. Yu P. C. Klompas M. Haessler S. D. Imrey P. B.. (2022). Bacterial coinfection in influenza pneumonia: Rates, pathogens, and outcomes. Infect. Control Hosp Epidemiol. 43, 212–217. doi: 10.1017/ice.2021.96 33890558 PMC9116507

[B6] Bastos M. D. R. de Figueiredo F. A. T. Macedo A. P. Silva A. C. F. Ferreira M. P. de Freitas O.. (2020). Local anesthetic improves individuals affected with herpes simplex type 1 labialis. J. Med. Virol. 92, 3638–3644. doi: 10.1002/jmv.25982 32374443

[B7] Batool S. Chokkakula S. Song M. S. (2023). Influenza treatment: limitations of antiviral therapy and advantages of drug combination therapy. Microorganisms 11 (1), 183. doi: 10.3390/microorganisms11010183 36677475 PMC9865513

[B8] Cassuto J. Sinclair R. Bonderovic M. (2006). Anti-inflammatory properties of local anesthetics and their present and potential clinical implications. Acta Anaesthesiol Scand. 50, 265–282. doi: 10.1111/j.1399-6576.2006.00936.x 16480459

[B9] Chai S. Zhan J. L. Zhao L. M. Liu X. D. (2022). Safety of triazole antifungals: a pharmacovigilance study from 2004 to 2021 based on FAERS. Ther. Adv. Drug Saf. 13, 20420986221143266. doi: 10.1177/20420986221143266 36545565 PMC9761248

[B10] Chan A. K. Y. Tsang Y. C. Chu C. H. Tsang C. S. P. (2021). Aspirin as an antifungal-lock agent in inhibition of candidal biofilm formation in surgical catheters. Infect. Drug Resist. 14, 1427–1433. doi: 10.2147/IDR.S308262 33888996 PMC8058035

[B11] Cramer R. A. Jr. Perfect B. Z. Pinchai N. Park S. Perlin D. S. Asfaw Y. G.. (2008). Calcineurin target CrzA regulates conidial germination, hyphal growth, and pathogenesis of *Aspergillus fumigatus* . Eukaryot Cell 7, 1085–1097. doi: 10.1128/EC.00086-08 18456861 PMC2446674

[B12] Czyrski A. Resztak M. Swiderski P. Brylak J. Glowka F. K. (2021). The overview on the pharmacokinetic and pharmacodynamic interactions of triazoles. Pharmaceutics 13(11), 1961. doi: 10.3390/pharmaceutics13111961 34834376 PMC8620887

[B13] Day A. M. Quinn J. (2019). Stress-activated protein kinases in human fungal pathogens. Front. Cell Infect. Microbiol. 9. doi: 10.3389/fcimb.2019.00261 PMC6652806 31380304

[B14] de Castro P. A. Chiaratto J. Winkelstroter L. K. Bom V. L. Ramalho L. N. Goldman M. H.. (2014). The involvement of the Mid1/Cch1/Yvc1 calcium channels in *Aspergillus fumigatus* virulence. PloS One 9, e103957. doi: 10.1371/journal.pone.0103957 25083783 PMC4118995

[B15] Duwe S. C. Schmidt B. Gartner B. C. Timm J. Adams O. Fickenscher H.. (2021). Prophylaxis and treatment of influenza: options, antiviral susceptibility, and existing recommendations. GMS Infect. Dis. 9, Doc02. doi: 10.3205/id000071 34113534 PMC8165743

[B16] Fox D. S. Heitman J. (2002). Good fungi gone bad: the corruption of calcineurin. Bioessays 24, 894–903. doi: 10.1002/bies.10157 12325122

[B17] Fuchs P. Levanon A. (1978). Inhibition of adsorption of West-Nile and herpes simplex viruses by procaine. Arch. Virol. 56, 163–168. doi: 10.1007/BF01317291 204270

[B18] Furuta Y. Komeno T. Nakamura T. (2017). Favipiravir (T-705), a broad spectrum inhibitor of viral RNA polymerase. Proc. Jpn Acad. Ser. B Phys. Biol. Sci. 93, 449–463. doi: 10.2183/pjab.93.027 PMC5713175 28769016

[B19] Furuta Y. Takahashi K. Kuno-Maekawa M. Sangawa H. Uehara S. Kozaki K.. (2005). Mechanism of action of T-705 against influenza virus. Antimicrob. Agents Chemother. 49, 981–986. doi: 10.1128/AAC.49.3.981-986.2005 15728892 PMC549233

[B20] Gregg K. S. Kauffman C. A. (2015). Invasive aspergillosis: epidemiology, clinical aspects, and treatment. Semin. Respir. Crit. Care Med. 36, 662–672. doi: 10.1055/s-0035-1562893 26398533

[B21] Hagiwara D. Suzuki S. Kamei K. Gonoi T. Kawamoto S. (2014). The role of AtfA and HOG MAPK pathway in stress tolerance in conidia of *Aspergillus fumigatus* . Fungal Genet. Biol. 73, 138–149. doi: 10.1016/j.fgb.2014.10.011 25459537

[B22] Häring C. Jungwirth J. Schroeder J. Loffler B. Engert B. Ehrhardt C. (2023). The local anaesthetic procaine prodrugs procCluster((R)) and procaine hydrochloride impair SARS-CoV-2 replication and egress *in vitro* . Int. J. Mol. Sci. 24(14), 584. doi: 10.3390/ijms241914584 PMC10572566 37834031

[B23] Häring C. Schroeder J. Jungwirth J. Löffler B. Henke A. Engert B.. (2024). ProcCluster® and procaine hydrochloride inhibit the replication of influenza A virus *in vitro* . Front. Microbiol. 15, 1422651. doi: 10.3389/fmicb.2024.1422651 39206370 PMC11350405

[B24] Häring C. Schroeder J. Loffler B. Engert B. Ehrhardt C. (2021). The local anaesthetic procaine prodrugs ProcCluster® and Procaine-hydrochloride impair SARS-CoV-2 replication *in vitro* . bioRxiv. doi: 10.1101/2021.06.07.447335 PMC10572566 37834031

[B25] Ianevski A. Giri A. K. Aittokallio T. (2022). SynergyFinder 3.0: an interactive analysis and consensus interpretation of multi-drug synergies across multiple samples. Nucleic Acids Res. 50, W739–W743. doi: 10.1093/nar/gkac382 35580060 PMC9252834

[B26] Iuliano A. D. Roguski K. M. Chang H. H. Muscatello D. J. Palekar R. Tempia S.. (2018). Estimates of global seasonal influenza-associated respiratory mortality: a modelling study. Lancet 391, 1285–1300. doi: 10.1016/S0140-6736(17)33293-2 29248255 PMC5935243

[B27] Joshi S. Parkar J. Ansari A. Vora A. Talwar D. Tiwaskar M.. (2021). Role of favipiravir in the treatment of COVID-19. Int. J. Infect. Dis. 102, 501–508. doi: 10.1016/j.ijid.2020.10.069 33130203 PMC7831863

[B28] Kaiserer L. Oberparleiter C. Weiler-Gorz R. Burgstaller W. Leiter E. Marx F. (2003). Characterization of the Penicillium chrysogenum antifungal protein PAF. Arch. Microbiol. 180, 204–210. doi: 10.1007/s00203-003-0578-8 12856109

[B29] Karakus U. Crameri M. Lanz C. Yángüez E. (2018). “Propagation and titration of influenza viruses,” in Methods Mol Biol. Influenza Virus: Methods and Protocols. Ed. Yamauchi Y. (Springer). 10.1007/978-1-4939-8678-1_4 30151569

[B30] König S. Schroeder J. Nietzsche S. Heinekamp T. Brakhage A. A. Zell R.. (2024). The influenza A virus promotes fungal growth of *Aspergillus fumigatus* via direct *interaction in vitro* . Microbes Infect. 26(3), 105264. doi: 10.1016/j.micinf.2023.105264 38008399

[B31] Koszalka P. Subbarao K. Baz M. (2022). Preclinical and clinical developments for combination treatment of influenza. PloS Pathog. 18, e1010481. doi: 10.1371/journal.ppat.1010481 35551301 PMC9098076

[B32] Kwon-Chung K. J. Sugui J. A. (2013). *Aspergillus fumigatus*–what makes the species a ubiquitous human fungal pathogen? PloS Pathog. 9, e1003743. doi: 10.1371/journal.ppat.1003743 24348239 PMC3857757

[B33] Lampejo T. (2020). Influenza and antiviral resistance: an overview. Eur. J. Clin. Microbiol. Infect. Dis. 39, 1201–1208. doi: 10.1007/s10096-020-03840-9 32056049 PMC7223162

[B34] Laniado-Laborin R. Cabrales-Vargas M. N. (2009). Amphotericin B: side effects and toxicity. Rev. Iberoam Micol 26, 223–227. doi: 10.1016/j.riam.2009.06.003 19836985

[B35] Lass-Flörl C. Dietl A. M. Kontoyiannis D. P. Brock M. (2021). *Aspergillus terreus* species complex. Clin. Microbiol. Rev. 34, e0031120. doi: 10.1128/CMR.00311-20 34190571 PMC8404697

[B36] Lass-Florl C. Griff K. Mayr A. Petzer A. Gastl G. Bonatti H.. (2005). Epidemiology and outcome of infections due to *Aspergillus terreus*: 10-year single centre experience. Br. J. Haematol 131, 201–207. doi: 10.1111/j.1365-2141.2005.05763.x 16197450

[B37] Lass-Flörl C. Nagl M. Speth C. Ulmer H. Dierich M. P. Wurzner R. (2001). Studies of *in vitro* activities of voriconazole and itraconazole against *Aspergillus* hyphae using viability staining. Antimicrob. Agents Chemother. 45, 124–128. doi: 10.1128/AAC.45.1.124-128.2001 11120954 PMC90249

[B38] Latge J. P. Chamilos G. (2019). *Aspergillus fumigatus* and aspergillosis in 2019. Clin. Microbiol. Rev. 33(1), e00140-18. doi: 10.1128/CMR.00140-18 31722890 PMC6860006

[B39] Ledoux M. P. Herbrecht R. (2023). Invasive pulmonary aspergillosis. J. Fungi (Basel) 9(2), 131. doi: 10.3390/jof9020131 36836246 PMC9962768

[B40] Lestrade P. P. Bentvelsen R. G. Schauwvlieghe A. Schalekamp S. van der Velden W. Kuiper E. J.. (2019). Voriconazole resistance and mortality in invasive aspergillosis: A multicenter retrospective cohort study. Clin. Infect. Dis. 68, 1463–1471. doi: 10.1093/cid/ciy859 30307492

[B41] Li X. Feng R. Luo P. Zhang Y. Lu L. (2024). Synergistic effects of putative Ca(2+)-binding sites of calmodulin in fungal development, temperature stress and virulence of *Aspergillus fumigatus* . Virulence 15, 2290757. doi: 10.1080/21505594.2023.2290757 38085844 PMC10761034

[B42] Li C. Gao S. Li X. Li C. Ma L. (2018). Procaine inhibits the proliferation and migration of colon cancer cells through inactivation of the ERK/MAPK/FAK pathways by regulation of RhoA. Oncol. Res. 26, 209–217. doi: 10.3727/096504017X14944585873622 28492141 PMC7844744

[B43] Li D. Gao J. Yang C. Li B. Sun J. Yu M.. (2021). cRGDyK-modified procaine liposome inhibits the proliferation and motility of glioma cells via the ERK/p38MAPK pathway. Exp. Ther. Med. 22, 859. doi: 10.3892/etm.2021.10291 34178132 PMC8220655

[B44] Lin T. Y. Chin C. R. Everitt A. R. Clare S. Perreira J. M. Savidis G.. (2013). Amphotericin B increases influenza A virus infection by preventing IFITM3-mediated restriction. Cell Rep. 5, 895–908. doi: 10.1016/j.celrep.2013.10.033 24268777 PMC3898084

[B45] Liu X. Zheng F. Tian L. Li T. Zhang Z. Ren Z.. (2023). Lidocaine inhibits influenza a virus replication by up-regulating IFNalpha4 via TBK1-IRF7 and JNK-AP1 signaling pathways. Int. Immunopharmacol 115, 109706. doi: 10.1016/j.intimp.2023.109706 36638664

[B46] Lother J. Breitschopf T. Krappmann S. Morton C. O. Bouzani M. Kurzai O.. (2014). Human dendritic cell subsets display distinct interactions with the pathogenic mould *Aspergillus fumigatus* . Int. J. Med. Microbiol. 304, 1160–1168. doi: 10.1016/j.ijmm.2014.08.009 25200858

[B47] Ludwig S. (2009). Targeting cell signalling pathways to fight the flu: towards a paradigm change in anti-influenza therapy. J. Antimicrob. Chemother. 64, 1–4. doi: 10.1093/jac/dkp161 19420020

[B48] Manfiolli A. O. Dos Reis T. F. de Assis L. J. de Castro P. A. Silva L. P. Hori J. I.. (2018). Mitogen activated protein kinases (MAPK) and protein phosphatases are involved in *Aspergillus fumigatus* adhesion and biofilm formation. Cell Surf 1, 43–56. doi: 10.1016/j.tcsw.2018.03.002 32743127 PMC7389341

[B49] May G. S. Xue T. Kontoyiannis D. P. Gustin M. C. (2005). Mitogen activated protein kinases of *Aspergillus fumigatus* . Med. Mycol 43 Suppl 1, S83–S86. doi: 10.1080/13693780400024784 16110797

[B50] Mazur I. Wurzer W. J. Ehrhardt C. Pleschka S. Puthavathana P. Silberzahn T.. (2007). Acetylsalicylic acid (ASA) blocks influenza virus propagation via its NF-kappaB-inhibiting activity. Cell Microbiol. 9, 1683–1694. doi: 10.1111/j.1462-5822.2007.00902.x 17324159

[B51] Millard P. J. Roth B. L. Thi H. P. Yue S. T. Haugland R. P. (1997). Development of the FUN-1 family of fluorescent probes for vacuole labeling and viability testing of yeasts. Appl. Environ. Microbiol. 63, 2897–2905. doi: 10.1128/aem.63.7.2897-2905.1997 9212436 PMC168585

[B52] Nagata T. Lefor A. K. Hasegawa M. Ishii M. (2015). Favipiravir: a new medication for the Ebola virus disease pandemic. Disaster Med. Public Health Prep 9, 79–81. doi: 10.1017/dmp.2014.151 25544306

[B53] Nair R. Raina S. Keshavarz T. Kerrigan M. J. (2011). Application of fluorescent indicators to analyse intracellular calcium and morphology in filamentous fungi. Fungal Biol. 115, 326–334. doi: 10.1016/j.funbio.2010.12.012 21530914

[B54] Neofytos D. Chatzis O. Nasioudis D. Boely Janke E. Doco Lecompte T. Garzoni C.. (2018). Epidemiology, risk factors and outcomes of invasive aspergillosis in solid organ transplant recipients in the Swiss Transplant Cohort Study. Transpl Infect. Dis. 20, e12898. doi: 10.1111/tid.12898 29668068

[B55] Obad J. Suskovic J. Kos B. (2015). Antimicrobial activity of ibuprofen: new perspectives on an "Old" non-antibiotic drug. Eur. J. Pharm. Sci. 71, 93–98. doi: 10.1016/j.ejps.2015.02.011 25708941

[B56] Pardo E. Lemiale V. Mokart D. Stoclin A. Moreau A. S. Kerhuel L.. (2019). Invasive pulmonary aspergillosis in critically ill patients with hematological Malignancies. Intensive Care Med. 45, 1732–1741. doi: 10.1007/s00134-019-05789-6 31599334

[B57] Patterson T. F. Thompson G. R. 3rd Denning D. W. Fishman J. A. Hadley S. Herbrecht R.. (2016). Practice guidelines for the diagnosis and management of aspergillosis: 2016 update by the infectious diseases society of America. Clin. Infect. Dis. 63, e1–e60. doi: 10.1093/cid/ciw326 27365388 PMC4967602

[B58] Pina-Vaz C. Sansonetty F. Rodrigues A. G. Costa-Oliveira S. Tavares C. Martinez-de-Oliveira J. (2001). Cytometric approach for a rapid evaluation of susceptibility of Candida strains to antifungals. Clin. Microbiol. Infect. 7, 609–618. doi: 10.1046/j.1198-743x.2001.00307.x 11737085

[B59] Pinchai N. Perfect B. Z. Juvvadi P. R. Fortwendel J. R. Cramer R. A. Jr. Asfaw Y. G.. (2009). *Aspergillus fumigatus* calcipressin CbpA is involved in hyphal growth and calcium homeostasis. Eukaryot Cell 8, 511–519. doi: 10.1128/EC.00336-08 19252123 PMC2669200

[B60] Renaud C. Kuypers J. Englund J. A. (2011). Emerging oseltamivir resistance in seasonal and pandemic influenza A/H1N1. J. Clin. Virol. 52, 70–78. doi: 10.1016/j.jcv.2011.05.019 21684202

[B61] Rodrigues A. G. Araujo R. Pina-Vaz C. (2006). Interaction of local anaesthetics with other antifungal agents against pathogenic *Aspergillus* . Int. J. Antimicrob. Agents 27, 339–343. doi: 10.1016/j.ijantimicag.2005.11.011 16527460

[B62] Rodrigues A. A. Pina-Vaz C. Mardh P. A. Martinez-de-Oliveira J. Freitas-da-Fonseca A. (2000). Inhibition of germ tube formation by Candida albicans by local anesthetics: an effect related to ionic channel blockade. Curr. Microbiol. 40, 145–148. doi: 10.1007/s002849910030 10679044

[B63] Roethl E. Gassner M. Krenn B. M. Romanovskaya-Romanko E. A. Seper H. Romanova J.. (2011). Antimycotic-antibiotic amphotericin B promotes influenza virus replication in cell culture. J. Virol. 85, 11139–11145. doi: 10.1128/JVI.00169-11 21849438 PMC3194987

[B64] Rudramurthy S. M. Paul R. A. Chakrabarti A. Mouton J. W. Meis J. F. (2019). Invasive aspergillosis by *aspergillus flavus*: epidemiology, diagnosis, antifungal resistance, and management. J. Fungi (Basel) 5(3), 55. doi: 10.3390/jof5030055 31266196 PMC6787648

[B65] Schauwvlieghe A. F. A. D. Rijnders B. J. A. Philips N. Verwijs R. Vanderbeke L. Van Tienen C.. (2018). Invasive aspergillosis in patients admitted to the intensive care unit with severe influenza: a retrospective cohort study. Lancet Respir. Med. 6, 782–792. doi: 10.1016/s2213-2600(18)30274-1 30076119

[B66] Schloer S. Goretzko J. Kuhnl A. Brunotte L. Ludwig S. Rescher U. (2019). The clinically licensed antifungal drug itraconazole inhibits influenza virus *in vitro* and *in vivo* . Emerg. Microbes Infect. 8, 80–93. doi: 10.1080/22221751.2018.1559709 30866762 PMC6455256

[B67] Seldeslachts L. Jacobs C. Tielemans B. Vanhoffelen E. van der Sloten L. Humblet-Baron S.. (2021a). Overcome double trouble: baloxavir marboxil suppresses influenza thereby mitigating secondary invasive pulmonary aspergillosis. J. Fungi (Basel) 8(1), 1. doi: 10.3390/jof8010001 35049941 PMC8777735

[B68] Seldeslachts L. Vanderbeke L. Fremau A. Resendiz-Sharpe A. Jacobs C. Laeveren B.. (2021b). Early oseltamivir reduces risk for influenza-associated aspergillosis in a double-hit murine model. Virulence 12, 2493–2508. doi: 10.1080/21505594.2021.1974327 34546839 PMC8923074

[B69] Shin W. J. Seong B. L. (2019). Novel antiviral drug discovery strategies to tackle drug-resistant mutants of influenza virus strains. Expert Opin. Drug Discovery 14, 153–168. doi: 10.1080/17460441.2019.1560261 30585088

[B70] Shiraki K. Daikoku T. (2020). Favipiravir, an anti-influenza drug against life-threatening RNA virus infections. Pharmacol. Ther. 209, 107512. doi: 10.1016/j.pharmthera.2020.107512 32097670 PMC7102570

[B71] Taubenberger J. K. Morens D. M. (2006). 1918 Influenza: the mother of all pandemics. Emerg. Infect. Dis. 12, 15–22. doi: 10.3201/eid1201.050979 16494711 PMC3291398

[B72] Tisi R. Rigamonti M. Groppi S. Belotti F. (2016). Calcium homeostasis and signaling in fungi and their relevance for pathogenicity of yeasts and filamentous fungi. AIMS Mol. Sci. 3, 505–549. doi: 10.3934/molsci.2016.4.505

[B73] Tobin J. M. Nickolich K. L. Ramanan K. Pilewski M. J. Lamens K. D. Alcorn J. F.. (2020). Influenza suppresses neutrophil recruitment to the lung and exacerbates secondary invasive pulmonary aspergillosis. J. Immunol. 205, 480–488. doi: 10.4049/jimmunol.2000067 32522833 PMC7416629

[B74] Ullmann A. J. Aguado J. M. Arikan-Akdagli S. Denning D. W. Groll A. H. Lagrou K.. (2018). Diagnosis and management of *Aspergillus* diseases: executive summary of the 2017 ESCMID-ECMM-ERS guideline. Clin. Microbiol. Infect. 24 Suppl 1, e1–e38. doi: 10.1016/j.cmi.2018.01.002 29544767

[B75] Vanderbeke L. Spriet I. Breynaert C. Rijnders B. J. A. Verweij P. E. Wauters J. (2018). Invasive pulmonary aspergillosis complicating severe influenza: epidemiology, diagnosis and treatment. Curr. Opin. Infect. Dis. 31, 471–480. doi: 10.1097/QCO.0000000000000504 30299367

[B76] van der Linden J. W. Arendrup M. C. Warris A. Lagrou K. Pelloux H. Hauser P. M.. (2015). Prospective multicenter international surveillance of azole resistance in *Aspergillus fumigatus* . Emerg. Infect. Dis. 21, 1041–1044. doi: 10.3201/eid2106.140717 25988348 PMC4451897

[B77] van der Linden J. W. Snelders E. Kampinga G. A. Rijnders B. J. Mattsson E. Debets-Ossenkopp Y. J.. (2011). Clinical implications of azole resistance in *Aspergillus fumigatus*, The Netherlands 2007-2009. Emerg. Infect. Dis. 17, 1846–1854. doi: 10.3201/eid1710.110226 22000354 PMC3311118

[B78] van de Veerdonk F. L. Kolwijck E. Lestrade P. P. Hodiamont C. J. Rijnders B. J. van Paassen J.. (2017). Influenza-associated aspergillosis in critically ill patients. Am. J. Respir. Crit. Care Med. 196, 524–527. doi: 10.1164/rccm.201612-2540LE 28387526

[B79] Wang J. L. Chang C. H. Young-Xu Y. Chan K. A. (2010). Systematic review and meta-analysis of the tolerability and hepatotoxicity of antifungals in empirical and definitive therapy for invasive fungal infection. Antimicrob. Agents Chemother. 54, 2409–2419. doi: 10.1128/AAC.01657-09 20308378 PMC2876415

[B80] Zhao X. Mehrabi R. Xu J. R. (2007). Mitogen-activated protein kinase pathways and fungal pathogenesis. Eukaryot Cell 6, 1701–1714. doi: 10.1128/EC.00216-07 17715363 PMC2043402

[B81] Zilberberg M. D. Harrington R. Spalding J. R. Shorr A. F. (2019). Burden of hospitalizations over time with invasive aspergillosis in the United States 2004-2013. BMC Public Health 19, 591. doi: 10.1186/s12889-019-6932-9 31101036 PMC6525423

