# Supplementary figures and images for: ProcCluster® and procaine hydrochloride inhibit the growth of Aspergillus species and exert antimicrobial properties during coinfection with influenza A viruses and A. fumigatus in vitro
Source: Front Cell Infect Microbiol. 2024 Oct 15;14:1445428. doi: 10.3389/fcimb.2024.1445428 (PMC11518849; doi:10.3389/fcimb.2024.1445428)

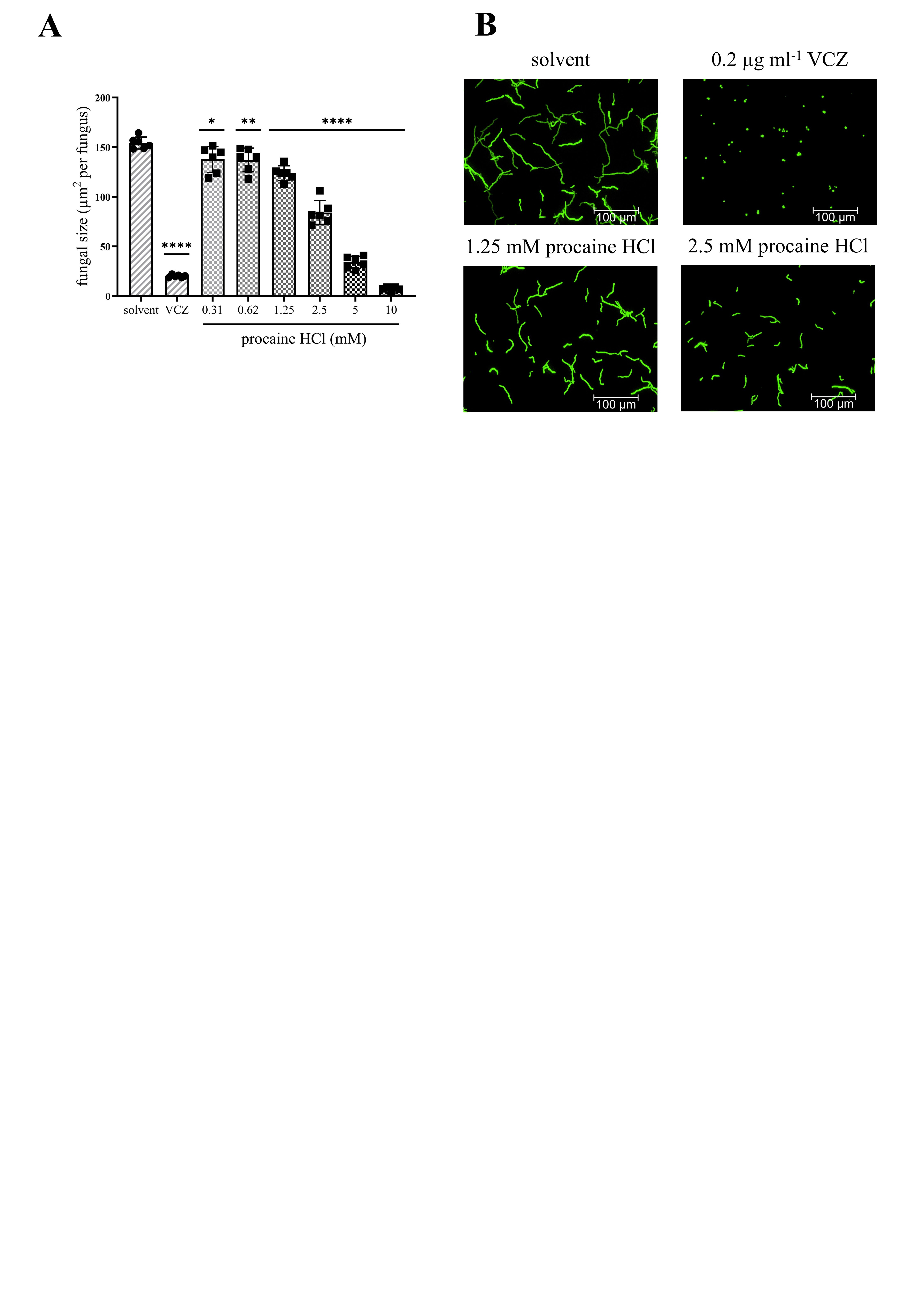

Supplement: Supplementary Figure 1 — In the presence of procaine HCl, the growth of A. fumigatus is efficiently inhibited in a human cell-free environment. Conidia of A. fumigatus were incubated with the indicated concentrations of procaine HCl, solvent (H2O) or 0.2 µg ml-1 VCZ for 10 h and growth of the fungus was quantified by fluorescence microscopy. (A) Quantification is given as µm2 per fungus. The diagram shows the mean (± SD) of the results of three independent experiments, including duplicates. (B) Selected fluorescence images showing GFP-expressing fungi treated with solvent (H2O), 0.2 µg ml-1 VCZ, 1.25 mM or 2.5 mM procaine HCl. Fluorescence images show one representative example of three independent experiments. (A) Statistical significance was assessed by one-way ANOVA followed by Dunnett’s multiple comparison test, comparing the mean of all samples with the solvent-treated sample (****p ≤ 0.0001, **p ≤ 0.01, *p ≤ 0.05). [file Image1.jpeg]

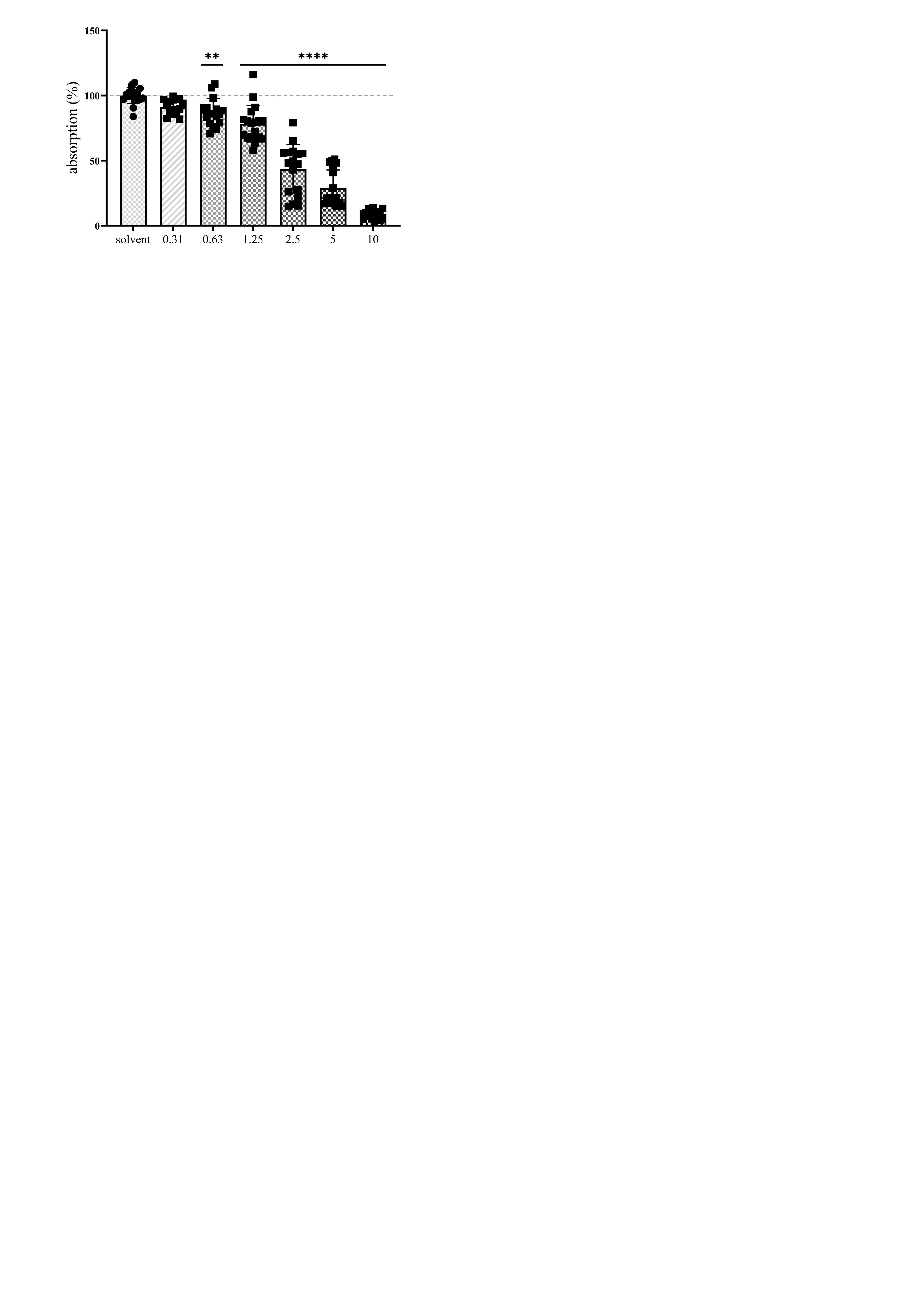

Supplement: Supplementary Figure 2 — Procaine HCl treatment affects the growth of A. fumigatus after 48 h. Conidia of A. fumigatus were treated with different concentrations of procaine HCl in a 96-well plate. After 48 h of incubation, the absorption of the cultures was measured at 620 nm. The graph shows the absorption in %, while the absorption of A. fumigatus growing in medium without drug or solvent was set to 100%. Diagram shows the mean (± SD) of the results from three independent experiments including 6 technical replicates. Statistical significance was assessed by one-way ANOVA followed by Dunnett’s multiple comparison test, comparing the mean of all samples with the solvent-treated sample (A) (****p ≤ 0.0001, **p ≤ 0.01). [file Image2.jpeg]

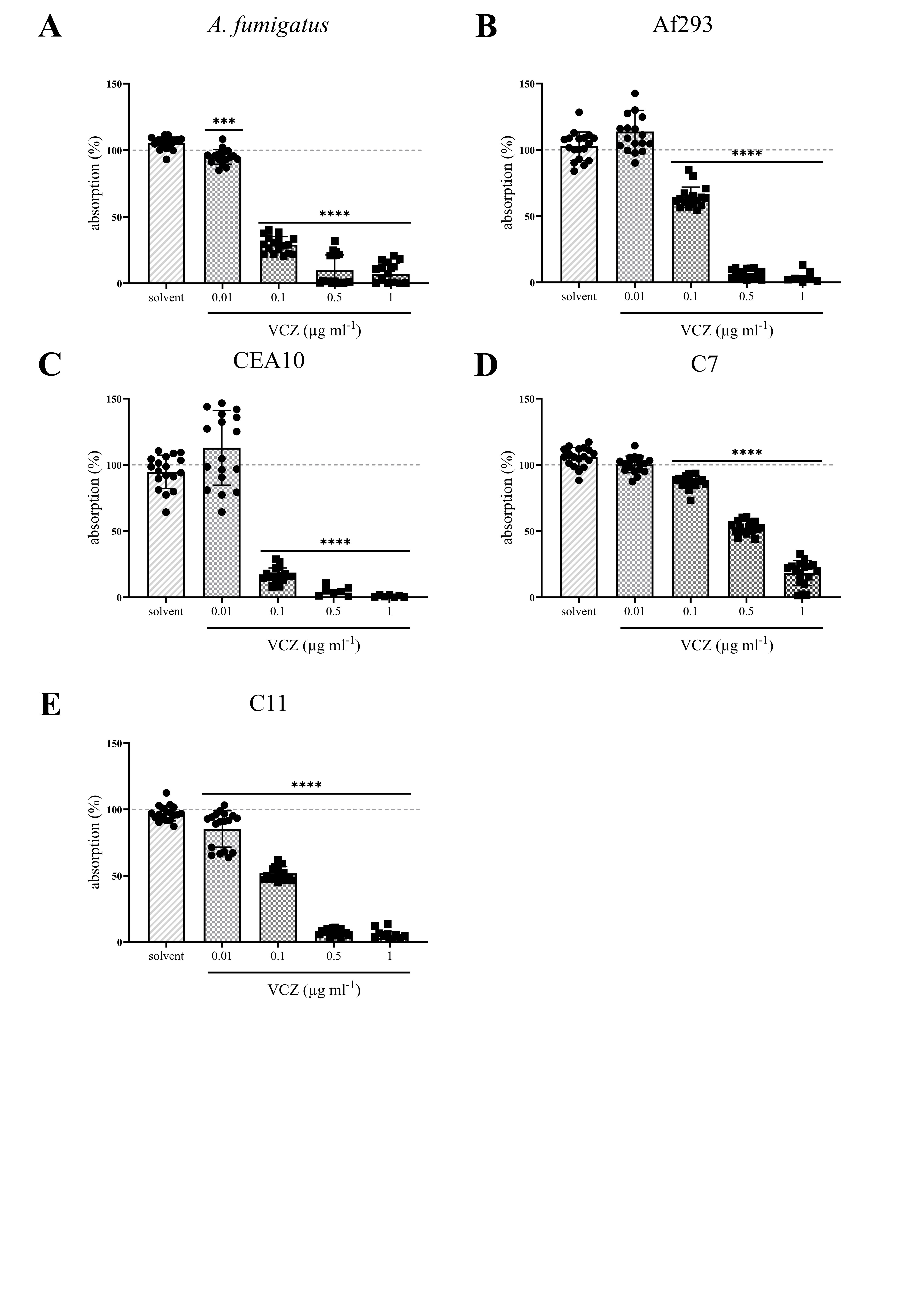

Supplement: Supplementary Figure 3 — A. fumigatus strain C7 is less sensitive to voriconazole treatment than other strains tested. Conidia of A. fumigatus (the strain used for most of the experiments) (A) and the A. fumigatus strains: CEA10 (B), Af293 (C), C7 (D) and C11 (E) were treated with different concentrations of VCZ or solvent (DMSO) in a 96-well plate. After 48 h of incubation, the absorbtion of the cultures was measured at 620 nm. Graphs show the absorption in %, while the absorption of A. fumigatus growing in medium without drug or solvent was set to 100%. Diagrams show the mean (± SD) of three independent experiments including 6 technical replicates. Statistical significance was assessed by one-way ANOVA followed by Dunnett’s multiple comparison test, comparing the mean of all samples with the solvent-treated sample (A) (****p ≤ 0.0001, ***p ≤ 0.001). [file Image3.jpeg]

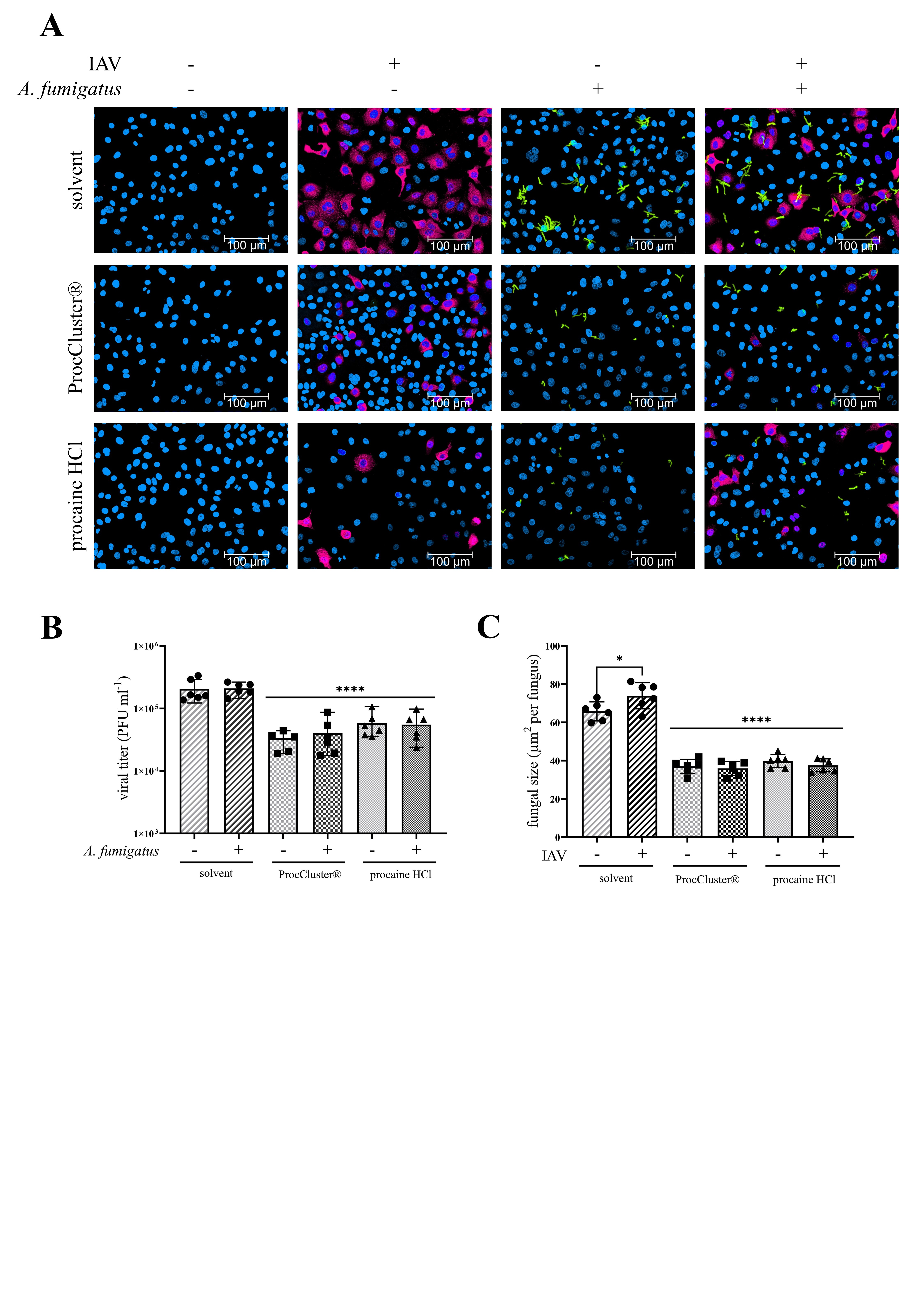

Supplement: Supplementary Figure 4 — ProcCluster® and procaine HCl have an antimicrobial effect on A. fumigatus and IAV during coinfection on A549 cells. (A–C) A549 cells were infected with IAV (H1N1; 0.5 MOI) for 30 min or left uninfected and were subsequently infected with conidia of A. fumigatus (10 MOI) for a further 10 h or left uninfected again. Concurrent the fungal infection, cells were treated with 2.5 mM ProcCluster®, procaine HCl or solvent (H2O). (A) Immunofluorescence images showing IAV-infected cells in red (stained with mouse anti-IAV-NP antibody and AlexaFluor674-conjugated donkey anti-mouse antibody), Hoechst 33342-stained nuclei of Calu-3 cells in blue and GFP-expressing A. fumigatus in green. All images were taken using an Axio Observer.Z1 microscope (Zeiss) at 20 × magnification. Scale bars represent 100 µm. The images show one representative example of three independent experiments. (B) Viral titers were analyzed using standard plaque assay. The graph shows the viral titer as PFU ml-1. (C) Immunofluorescence images were also used to quantify the growth (µm2 per fungus) of A. fumigatus in the presence and absence of the tested drugs. (B, C) Graphs show the mean (± SD) of the results of three independent experiments including duplicates. Statistical significance was assessed by one-way ANOVA followed by Tukey’s multiple comparison test. Statistical significances shown in (B, C) refer to the comparison between ProcCluster® or procaine HCl-treated samples and the corresponding solvent-treated control (****p ≤ 0.0001, *p ≤ 0.05). [file Image4.jpeg]

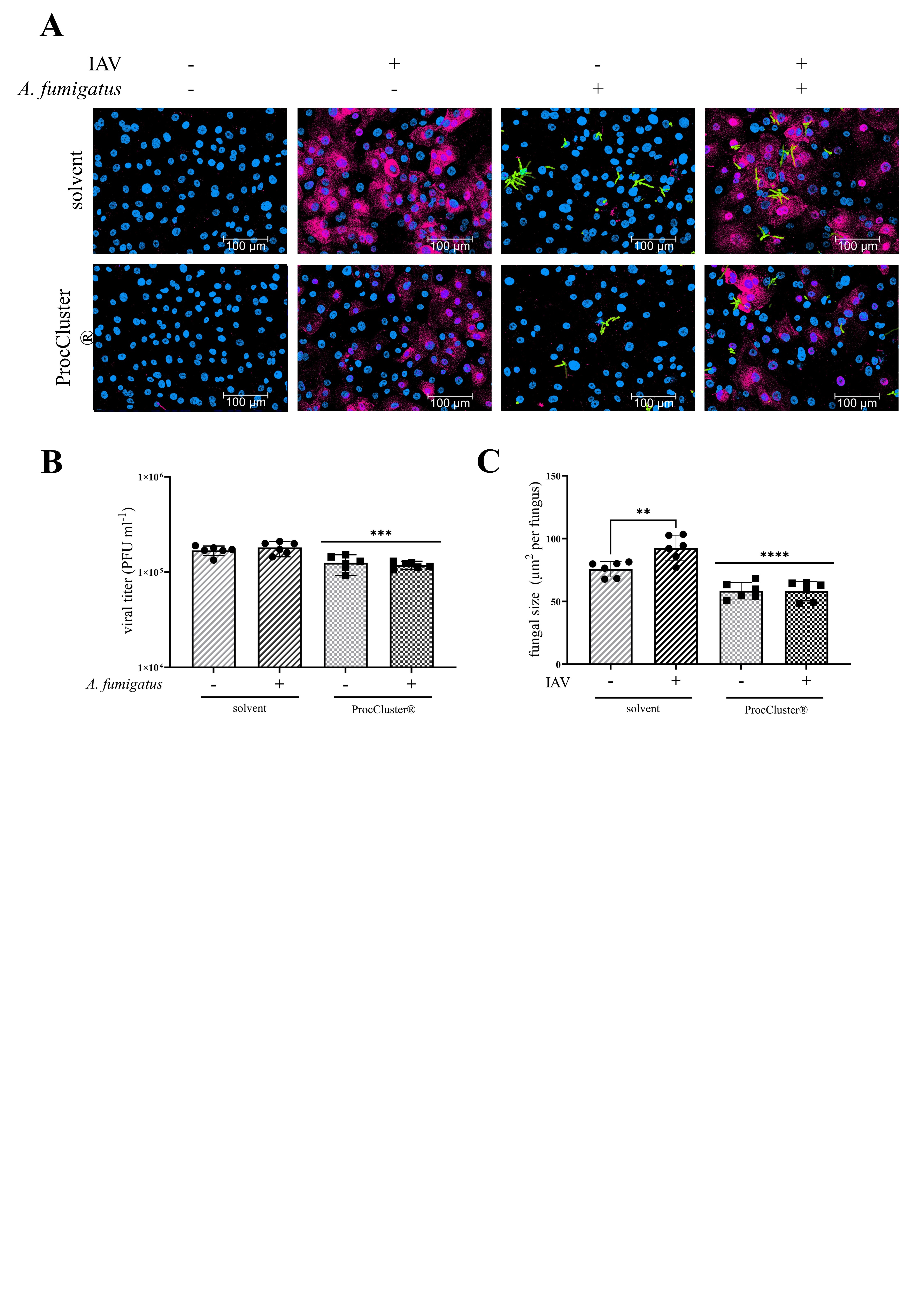

Supplement: Supplementary Figure 5 — ProcCluster® has an antimicrobial effect on A. fumigatus and IAV during coinfection on HBEpCs. HBEpCs were infected with IAV (H1N1; 5 MOI) for 30 min or left uninfected and were subsequently infected with conidia of A. fumigatus (10 MOI) for further 10 h or were left uninfected again. Concurrent with fungal infection, cells were treated with 2.5 mM ProcCluster® or with solvent (H2O). (A) Immunofluorescence images showing IAV-infected cells in red (stained with mouse anti-IAV-NP antibody and AlexaFluor674-conjugated donkey anti-mouse antibody), Hoechst 33342-stained nuclei of Calu-3 cells in blue and GFP-expressing A. fumigatus in green. All images were taken using an Axio Observer.Z1 microscope (Zeiss) at 20 × magnification. Scale bars represent 100 µm. The images show one representative example of three independent experiments. (B) Viral titers of IAV were analyzed using standard plaque assay. The graph shows the viral titer as PFU ml-1. (C) Immunofluorescence images were also used to quantify the growth (µm2 per fungus) of A. fumigatus in the presence and absence of the tested drugs. (B, C) Diagrams show the mean (± SD) of the results of three independent experiments, including duplicates. Statistical significance was evaluated by one-way ANOVA followed by Tukey’s multiple comparison test. Statistical significances shown in (B, C) refer to the comparison between ProcCluster® or procaine HCl-treated samples and the appropriate solvent-treated control (****p ≤ 0.0001, ***p ≤ 0.001, **p ≤ 0.01). [file Image5.jpeg]

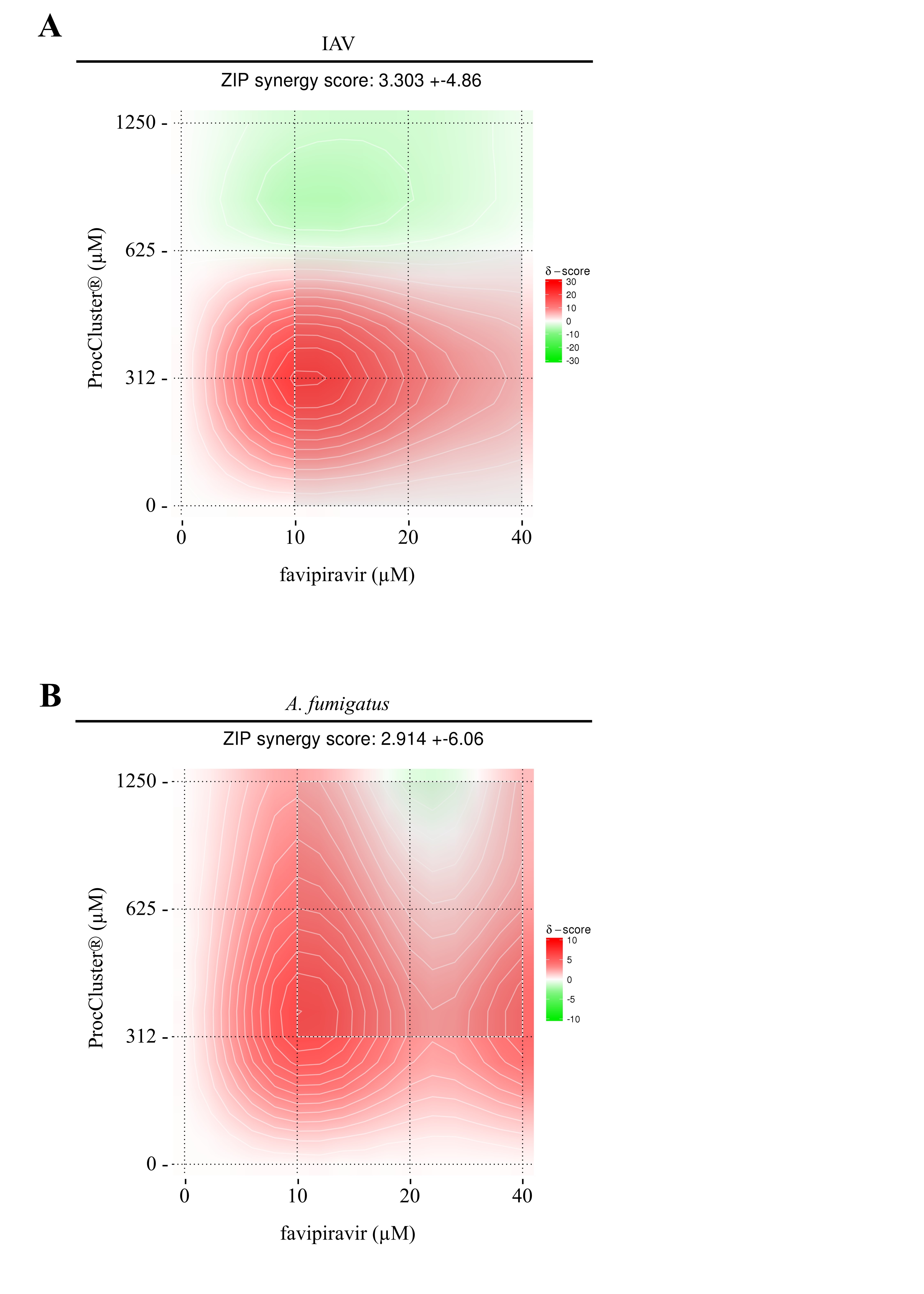

Supplement: Supplementary Figure 6 — Combination treatment with ProcCluster® and favipiravir has slight additive inhibitory effects against IAV and A. fumigatus during coinfection in vitro. The raw data of IAV titers (A) and hyphal growth of A. fumigatus (B), shown in Figure 6 were further analyzed using SynergyFinder 3.0 with the synergy model zero interaction potency (ZIP) and are shown as 2D plots generated by SynergyFinder. [file Image6.jpeg]

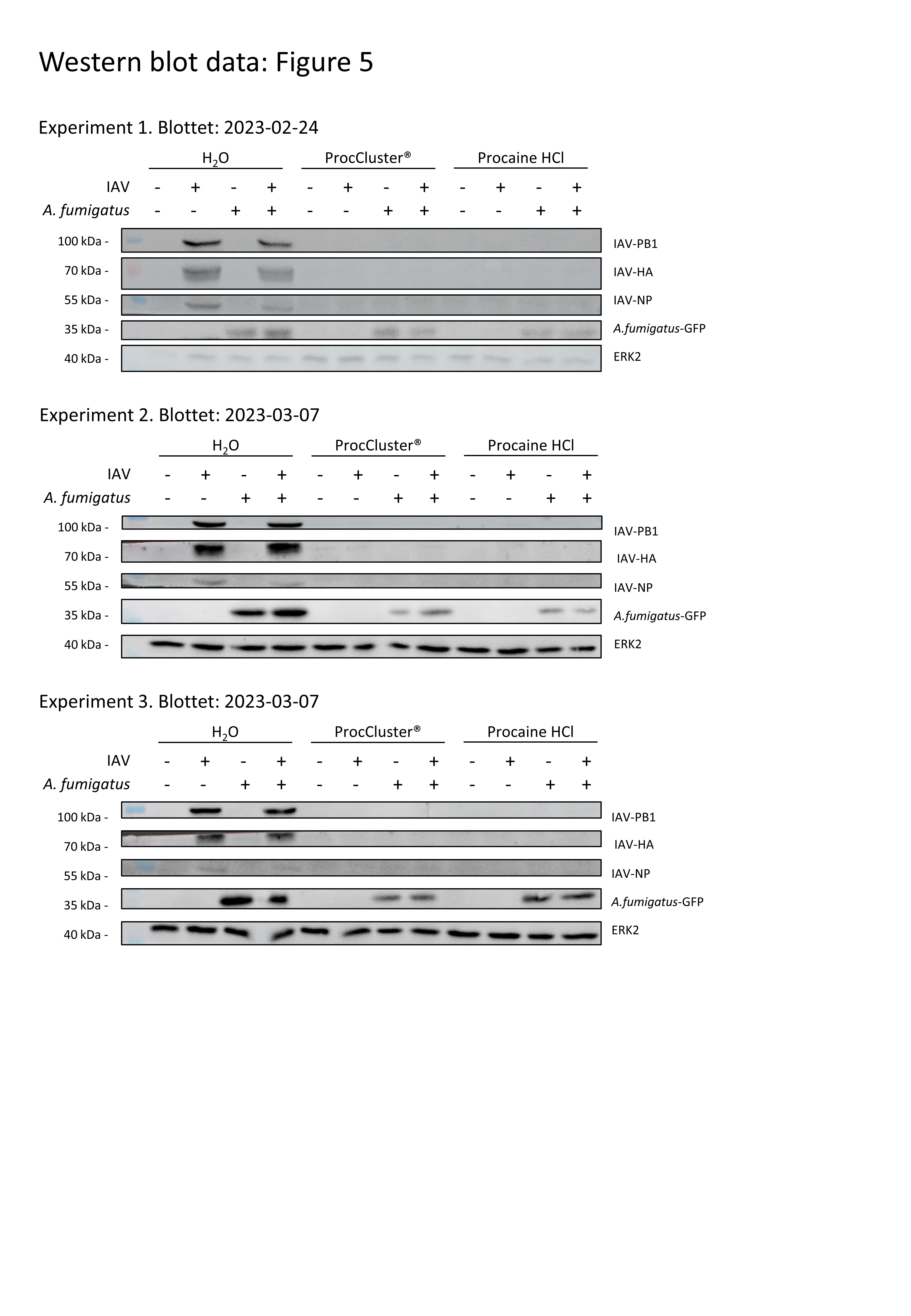

Supplement: Supplementary file 7 [file Image7.jpeg]
